# A snapshot of the practicality and barriers to COVID-19 interventions: Public health and healthcare workers’ perceptions in high and low- and middle-income countries

**DOI:** 10.1371/journal.pone.0260041

**Published:** 2021-11-24

**Authors:** Rosanna Glazik, Hannah Moore, David Kennedy, Hilary Bower, Hana Rohan, Ashley Sharp, Anna C. Seale

**Affiliations:** 1 UK Public Health Rapid Support Team, Public Health England/London School of Hygiene & Tropical Medicine, London, United Kingdom; 2 UK Field Epidemiology Training Programme (FETP), Public Health England, London, United Kingdom; 3 Department of Infectious Disease Epidemiology, London School of Hygiene & Tropical Medicine, London, United Kingdom; Xiamen University - Malaysia Campus, MALAYSIA

## Abstract

**Background:**

In response to the COVID-19 pandemic, governments have implemented a range of non-pharmaceutical interventions (NPIs) and pharmaceutical interventions (PIs) to reduce transmission and minimise morbidity and mortality, whilst maintaining social and economic activities. The perceptions of public health workers (PHWs) and healthcare workers (HCWs) are essential to inform future COVID-19 strategies as they are viewed as trusted sources and are at the forefront of COVID-19 response. The objectives of this study were to 1) describe the practicality of implementing NPIs and PIs and 2) identify potential barriers to implementation, as perceived by HCWs and PHWs.

**Methods:**

We conducted a cross-sectional study of PHWs and HCWs perceptions of the implementation, practicality of, and barriers to implementation of NPIs and PIs using an online survey (28/9/2020-1/11/2020) available in English, French and Portuguese. We used descriptive statistics and thematic analysis to analyse quantitative and qualitative responses.

**Results:**

In total, 226 respondents (67 HCWs and 159 PHWs) from 52 countries completed the survey and 222 were included in the final analysis. Participants from low and middle-income countries (LMICs) accounted for 63% of HCWs and 67% of PHWs, with the remaining from high-income (HICs). There was little difference between the perceptions of PHWs and HCWs in HICs and LMICs, with the majority regarding a number of common NPIs as difficult to implement. However, PHWs in HICs perceived restrictions on schools and educational institutions to be more difficult to implement, with a lack of childcare support identified as the main barrier. Additionally, most contact tracing methods were perceived to be more difficult to implement in HICs than LMICs, with a range of barriers reported. A lack of public support was the most commonly reported barrier to NPIs overall across both country income and professional groups. Similarly, public fear of vaccine safety and lack of vaccine supply were the main reported barriers to implementing a COVID-19 vaccine. However, PHWs and HCWs in LMICs perceived a lack of financial support and the vaccine being manufactured in another country as additional barriers.

**Conclusion:**

This snapshot provides insight into the difficulty of implementing interventions as perceived by PHWs and HCWs. There is no one-size-fits-all solution to implementing interventions, and barriers in different contexts do vary. Barriers to implementing a vaccine programme expressed here by HCWs and PHCWs have subsequently come to the fore internationally.

## Introduction

Since the WHO declared COVID-19 a global pandemic on the 11^th^ March 2020 [1], governments have sought to balance reducing transmission with maintaining social and economic activities. Governments have responded by implementing a range of non-pharmaceutical interventions (NPIs) and pharmaceutical interventions (PIs) to reduce transmission and minimise morbidity and mortality [2]. NPIs have included travel restrictions, stay at home orders, closure of schools, and individual measures, such as the use of face masks and improved hand hygiene. PIs have included treatments for disease and vaccines for prevention. However, there is increasing evidence highlighting significant direct and indirect health, economic and societal effects of interventions across different contexts [3–9]. In other epidemics, we have seen measures implemented with greater consequences for the people living in those contexts, particularly in countries with less resources [8, 10]. With the pandemic still evolving, it is clear that NPIs need to be implemented to complement vaccines. However, there is no one-size-fits-all approach [11], and whilst more evidence has emerged about public perceptions of PIs and their effectiveness, more is required on NPIs, including a deeper understanding of barriers to implementation and enabling factors for adherence in different populations [12, 13].

Different countries have implemented NPIs and PIs with varying levels of success [14–18]. Socioeconomic and cultural disparities across the world have affected the implementation of COVID-19 interventions, including vaccines [19, 20]. Furthermore, public perceptions and responses to interventions have differed across contexts throughout the pandemic [21–24]. Public trust is key for ensuring policy adherence during epidemics and pandemics and reports suggest that healthcare workers (HCWs) and public health workers (PHWs) are more trusted than other sources, including government [25–28]. However, whilst HCWs and PHWs may be more trusted, evidence has highlighted several factors which may impede their ability to influence public engagement with or adherence to interventions [25, 29–31]. For example, underuse of HCWs in providing public health education, HCWs not having a platform to express their opinions, a lack of recognition of their role in decision-making and the politicization of science. There is limited research focused on assessing the perceptions of COVID-19 interventions of PHWs and HCWs [32–36] and most related literature is centered on public opinions. With PHWs and HCWs at the forefront of COVID-19 response, it is important that their perceptions inform future preparedness and response strategies [31, 37].

This study aimed to provide insight into HCW and PHW perceptions of the practicality of and potential barriers to implementation and adherence to NPIs and PIs across the world. Although the data presented here were from a small sample across different countries, they provide an important descriptive indication of the perceptions of these trusted groups, and will assist decision-makers by suggesting which interventions are more likely to be accepted and/or feasible in different contexts now and in the future.

## Methods

### Study design

This was a cross-sectional observational study conducted using an online survey (https://www.onlinesurveys.ac.uk/) to collect quantitative and qualitative data on PHWs’ and HCWs’ perceptions of the implementation, practicality of, and barriers to implementation of NPIs and PIs. Data collection was performed using the Online Surveys platform [38]. An invitation to complete the survey was shared with professional and personal networks via email, social media, mobile applications, and other platforms. A snowball sampling technique was adopted to recruit subjects, whereby networks and survey participants were asked to share the survey with other relevant networks.

### Data collection

Separate online surveys for PHWs and HCWs were piloted for one week with PHWs and HCWs from HICs and LMICs to assess the validity and reliability. Questions and response options were refined and the surveys were fully opened for participation from the 28th September 2020 to the 1^st^ November 2020. The inclusion criteria for participants were: 1) PHW, defined as professionals engaged in public health activities, or HCW, defined as professionals who work in healthcare or social care settings; 2) aged over 18 years; 3) consent to take part in the study. To obtain informed consent, a checkbox for the participant to tick to agree to take part in the study was included in the survey. The surveys were disseminated through email, social media, mobile applications, and other online platforms. A short description of the study and links to the surveys were provided. The surveys were available in English, French and Portuguese. Contacted networks were asked to share this information with other relevant networks using the same methods.

### Study survey

Questions were developed based on key COVID-19 NPIs and PIs (Table 1) [2]. To recognise the different professional roles and responsibilities of PHWs and HCWs in these interventions, specific questions were directed only to PHWs and others to HCWs. PHWs and HCWs answered the same questions on shielding and vaccines as it was assumed both would potentially have some involvement with these interventions. At the time of the survey, no vaccines had been approved for use, so respondents were asked to answer questions on vaccine use hypothetically, assuming a safe and effective vaccine for COVID-19 could be developed. For each NPI and PI, respondents were asked 1) if they had been implemented and what they were specifically, 2) how easy did they think it was to implement the intervention, and 3) what they perceived as the barriers to implementation. The survey included routing, so when participants indicated the intervention was not implemented, they would not answer questions on the practicality of implementing interventions. Similarly, respondents only answered questions on the barriers to implementation if they perceived the intervention as “Difficult” or “Very difficult” to implement. HCWs were also asked about essential healthcare services to provide insights on the perceived impact of COVID-19 on these services.

**Table 1:** Non-pharmaceutical and pharmaceutical interventions assessed in the surveys.

	Public Health Workers	Healthcare Workers
**NPIs**	Shielding*	
	Travel restrictions	Personal Protective Equipment (PPE)
	Restrictions on offices, businesses and institutions	
	Restrictions on gatherings	
	Individual measures (e.g. handwashing, facemasks)	
	Contact tracing	
**PIs**	Vaccines	
		Therapeutics
**Other**		Essential Healthcare Services
	Demographics	

* Shielding was defined in the survey as a measure to protect vulnerable people from coming into contact with COVID-19, by restricting interactions between them and others [39].

The majority of the questions were multiple choice with pick one/many option(s) and free text space if “Other” was selected. There was no limit to the amount of text a respondent could insert. Likert scale questions were used to evaluate the practicality of implementing interventions, with options including “Very easy”, “Easy”, “Difficult” and “Very difficult”. All questions were mandatory to complete but included a “Don’t know” option.

Participants were asked to answer questions based on their professional opinion with respect to the country they are most associated with, for example, where they work or where they live at the time of completing the survey.

Basic demographic information (including age, gender, country and occupation) was collected to describe the respondents, and this was also used to identify differences in responses between healthcare and public health workers.

### Data analysis

Quantitative data were analysed using the statistical software R version 4.0.1. Descriptive analysis (frequencies/percentages) was performed for each question for high-income countries (HICs) and low and middle income countries (LMICs). Countries were classified into HICs and LMICs based on the World Bank country income classification [40] and classified into WHO regions to capture geographical distribution of respondents [41]. Where the number of responses for a category or country group was more than four, p-values were calculated using a χ^2^ test to determine any statistically significant difference between the responses. For questions where the number of responses was four or less, a Fisher’s exact test was used instead. P-values were not calculated if there were no responses in a category or country grouping and for “Other” and “Don’t know” responses to aid interpretation.

Responses to the questions on the practicality of implementing interventions were analysed by grouping responses into the following two groups to summarise the results: “Very easy”/ “Easy” and “Difficult”/ “Very difficult”. Where respondents did not provide the name of the country they were answering the survey for, their responses were excluded from the analysis. For the comparison of the practicality of implementing interventions and the country classification groups, only “Easy” or “Very Easy” and “Difficult” or “Very difficult” categories were included in the comparison. “Other” and “Don’t know” responses were excluded to aid interpretation. Where there were no responses for either HICs or LMICs, p-values were not calculated.

Qualitative data were analysed by translating the French and Portuguese text into English and subsequently performing a thematic analysis. Two authors independently used an interpretative approach to identify common themes. The authors triangulated these themes with the quantitative results to triangulate the findings.

### Ethical approval

This study was approved by the London School of Hygiene & Tropical Medicine (LSHTM) Ethics Committee (Reference: 21872). Participants gained access to the survey after providing written consent on the online survey platform. The surveys were anonymous, therefore no information that could identify participants was collected or stored. Participants voluntarily provided email addresses if they wished to be contacted at a later stage regarding future research work on COVID-19.

## Results

### Participants

There were 226 respondents from 52 countries, 67 HCWs and 159 PHWs. 4 PHW respondents were excluded from the analysis as they did not indicate which country they answered the survey questions for.

### Demographics

Respondents aged 35–44 comprised the largest proportion of participants, and 52% of both occupational categories were male (Table 2). Participants from LMICs accounted for 63% of HCWs and 67% PHWs, with the remaining from HICs. There was a greater representation of respondents from the WHO Africa and Europe regions. The majority of both HCW and PHW respondents (50%) were government employees (Table 2).

**Table 2:** Respondents’ demographics.

	Healthcare Workers (HCWs) n = 67	Public Health Workers (PHWs) n = 155
**Gender**		
Male	52% (n = 35)	52% (n = 80)
Female	46% (n = 31)	45% (n = 70)
Prefer not to say	2% (n = 1)	3% (n = 5)
**Age**		
18–34	27% (n = 18)	28% (n = 44)
35–44	33% (n = 22)	36% (n = 56)
45–54	24% (n = 16)	16% (n = 24)
55–64	12% (n = 8)	12% (n = 19)
65–75+	3% (n = 2)	6% (n = 9)
Prefer not to say	1% (n = 1)	12% (n = 3)
**Country type**		
High-income country (HIC)	37% (n = 25)	33% (n = 51)
Low or middle-income country (LMIC)	63% (n = 42)	67% (n = 104)
**WHO Region**		
Africa	52% (n = 35)	50% (n = 77)
Eastern Mediterranean	1% (n = 1)	8% (n = 12)
Europe	33% (n = 22)	26% (n = 41)
Americas	6% (n = 4)	9%(n = 14)
South-East Asia	7% (n = 5)	5% (n = 8)
Western Pacific	-	2% (n = 3)
**Type of institution** *		
Government	-	47% (n = 72)
Non-governmental or not-for-profit agency	-	19% (n = 30)
Research	-	16% (n = 25)
Higher Education	-	16% (n = 25)
Other	-	5% (n = 8)
Multilateral organisation	-	3% (n = 6)
Government referral hospital	27% (n = 18)	-
Other	25% (n = 17)	-
Community clinic/health post	19% (n = 13)	-
Research institute	16% (n = 11)	-
Government local/district hospital	13% (n = 9)	-
Private community clinic	6% (n = 4)	-
Private local/district hospital	6% (n = 4)	-
Private referral hospital	1% (n = 1)	-
**Profession**		
Doctor	52% (n = 35)	-
Other	22% (n = 15)	-
Nurse	21% (n = 14)	-
Community health worker	4% (n = 3)	-
**Work level** *		
National	-	49% (n = 76)
International	-	25% (n = 39)
Community/District	-	19% (n = 30)
County/Regional	-	15% (n = 24)
Other	-	3% (n = 4)

* Respondents could select more than one answer.

### Assessment of non-pharmaceutical interventions

**Fig 1:**
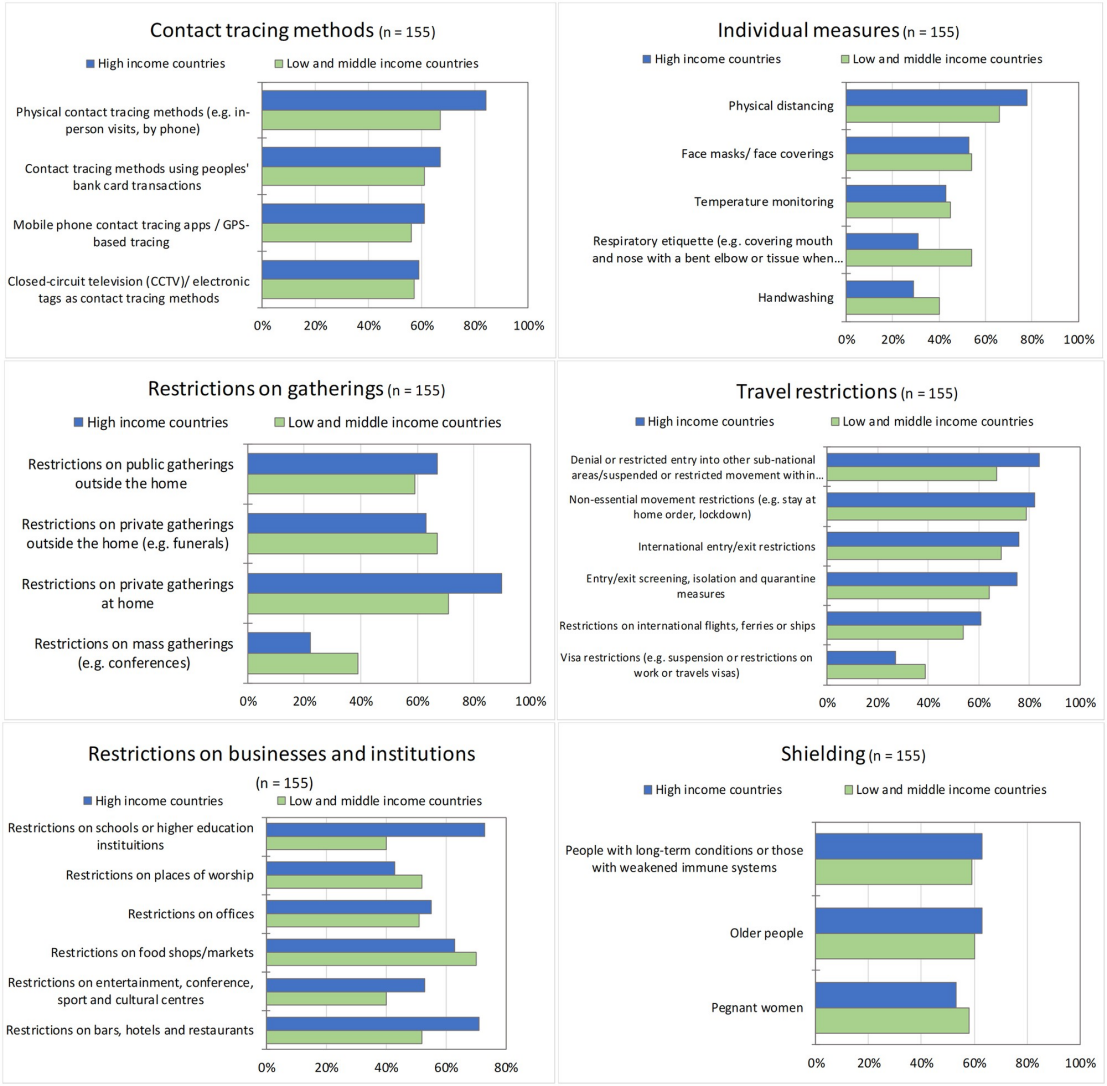
Percentage of respondents rating the following interventions as the most difficult to implement, grouped by intervention type.

Reported interventions implemented are shown in S1 Table in S1 File. Fig 1 outlines the interventions reported as the most difficult to implement for both HICs and LMICs.

#### Travel restrictions

Travel restrictions were perceived as the most difficult type of restrictions to implement overall (S2 Table in S1 File). Domestic travel restrictions were reported to be the most difficult to implement by respondents in both LMICs and HICs, with non-essential movement restrictions perceived as the most difficult to implement (HICs = 82% and LMICs = 79%).

A lack of public support was a commonly reported barrier for implementing these measures by all PHWs, particularly for implementing non-essential movement restrictions (S3 Table in S1 File). A lack of capacity to enforce also emerged as a major barrier to implementing these measures for both country income groups (HICs = 61% and LMICs = 53%), in addition to other measures, such as international entry/exit restrictions and entry/exit screening, isolation and quarantine measures.

Limited cross-country collaboration and corruption were also noted repeatedly as barriers to implementing several travel restrictions, particularly with regards to land borders. As one respondent noted, “*Corruption and bribery is rife and therefore some of these restrictions go unheeded*. *Border squabbles also contribute to these challenges on the political scene” [PHW*, *LMIC]*. Qualitative responses also highlighted concerns around the evidence to support travel restrictions, in particular, domestic restrictions, in addition to the challenges of monitoring adherence to these measures, and the impact of these restrictions, on livelihoods, and noted the lack of income support:

“*We had large numbers of people who needed to move between regions because they had no means to stay where they were*. *This was not considered in the lockdown*, *but it was a huge social problem that turned into a health problem*”.

*[PHW*, *LMIC]*.

“*Trade*, *and livelihoods*, *are now internationally dependent*. *Limiting travel leads to poverty*”.

*[PHW*, *LMIC]*.

#### Restrictions on offices, businesses and institutions

There were differences in the perceived ease of implementing restrictions on offices, businesses and institutions across the country income groups. In general, they were perceived to be more difficult to implement by PHWs in HICs. In particular, PHWs in HICs perceived restrictions on schools and educational institutions as “difficult” or “very difficult” to implement (70%) compared to PHWs in LMICs (40%).

Respondents in HICs reported a lack of public support as the most common barrier for implementing restrictions on almost all businesses and institutions listed (26%–57%), followed by a lack of financial support (S4 Table in S1 File). Similar barriers were perceived by PHWs in LMICs. Barriers to implementing restrictions on schools and higher education institutes were reported by many respondents, particularly in HICs, where a lack of childcare support was reported by more than 60% of participants. Qualitative responses from PHWs in LMICs and HICs suggested the impact of school closures on child development and mental health is also a key barrier: *“Keeping education open is an imperative—the barrier would be in instituting restrictions in the first place” [PHW*, *HIC]*.

#### Restrictions on gatherings

Respondents in both HIC and LMIC groups perceived restrictions on private gatherings at home as the most difficult to implement; 90% and 71% respectively.

The main barriers to implementing restrictions on all types of gatherings were perceived to be a lack of public support and a lack of enforcement capacity among both country income groups (S5 Table in S1 File). A lack of communication between policy-makers and the public, and socio-cultural practices and beliefs were identified as further barriers to restricting private gatherings inside/outside of homes.

#### Individual measures

Physical distancing was almost universally perceived as the most difficult individual measure to implement by 78% of respondents in HICs and 66% of respondents in LMICs.

A wide range of barriers to implementing individual measures were reported in similar proportions among both country income groups (S6 Table in S1 File). Respondents in LMICs reported a greater number of barriers overall; most frequently, a lack of resources, misinformation and socio-cultural beliefs and practices. The main barriers among PHWs in HICs were a lack of enforcement capacity and public support. A lack of public support was the most commonly cited barrier for physical distancing and face masks/coverings by PHWS in both LMICs and HICs. Qualitative responses identified a lack of public understanding due to insufficient information as an additional barrier to implementing face masks/ coverings and physical distancing. Several respondents also reported a lack of evidence as a barrier to implementing face masks/coverings and temperature monitoring. Qualitative responses also support socio-cultural beliefs and practices and limited risk perception as barriers to implementing physical distancing, particularly among respondents in LMICs:

“*The major problem was habitual and cultural adaptation*. *It was something new for societies and requires a lot of efforts from government and public sides to accept the new reality*”

*[PHW*, *LMIC]*.

“*If COVID was like Ebola*, *I think it would be supported but most people have no personal or family experience of COVID illness and are unconvinced by the necessity of physical distancing*”

*[PHW*, *LMIC]*.

#### Contact tracing

The majority of respondents perceived most contact tracing methods to be difficult to implement; however, respondents in HICs perceived them to be more difficult than respondents in LMICs. Contact tracing using people’s bank card transactions was reported to be the most difficult method to implement; PHWs in LMICs highlighted that most transactions are in cash, meaning contact tracing by bank card transactions would not be feasible.

Barriers for physical contact tracing (e.g. in-person visits, by phone) predominantly included lack of trained staff, lack of public support and lack of technology infrastructure for both HICs and LMICs (S7 Table in S1 File). For other contact tracing methods, such as closed-circuit television (CCTV)/ electronic tags and mobile phone contact tracing apps / GPS-based tracing, respondents from HICs most commonly cited privacy laws and lack of public support among top barriers, whereas respondents from LMICs reported lack of technology infrastructure, lack of trained staff and lack of public support. Additional barriers identified through qualitative responses included the reliability of some methods, including physical contact tracing and mobile phone contact tracing apps /GPS-based tracing:

“*Misinformation by contact tracers plus poor performance in tracing people means the public have lost trust in the system*”

*[PHW*, *HIC]*.

*Shielding of vulnerable populations*. The majority of participants, regardless of income group or job role, rated the overall ease of implementing shielding—defined in the survey as a measure to protect vulnerable people from coming into contact with COVID-19, by restricting interactions between them and others [39]—as “difficult” or “very difficult”.

Reported barriers to shielding pregnant women, older people and people with long-term conditions were similar across these three population group categories but differed between participants in HICs and LMICs (S8 Table in S1 File). A lack of home, financial or public support were the most commonly reported barriers among both country groups. Additionally, the mental health impact of shielding was identified as a key barrier across these three population groups by both PHWs and HCWs in LMICs and HICs:

“*Social isolation—some may choose to take COVID related risks rather than risk mental/social health*”

*[HCW*, *HIC]*.

HCWs in both country income groups also highlighted that people with long-term conditions or those with weakened immune systems need to continue to attend appointments at healthcare facilities, indicating that this is a further barrier to implementing shielding measures for this population group:

*Majority of the […] population access health care through the public sector*. *They therefore have to visit clinics and hospitals which are high-risk areas due to the number of people attending those facilities on a particular day*”

*[HCW*, *LMIC)*.

### Assessment of pharmaceutical interventions

#### Vaccines

**Fig 2:**
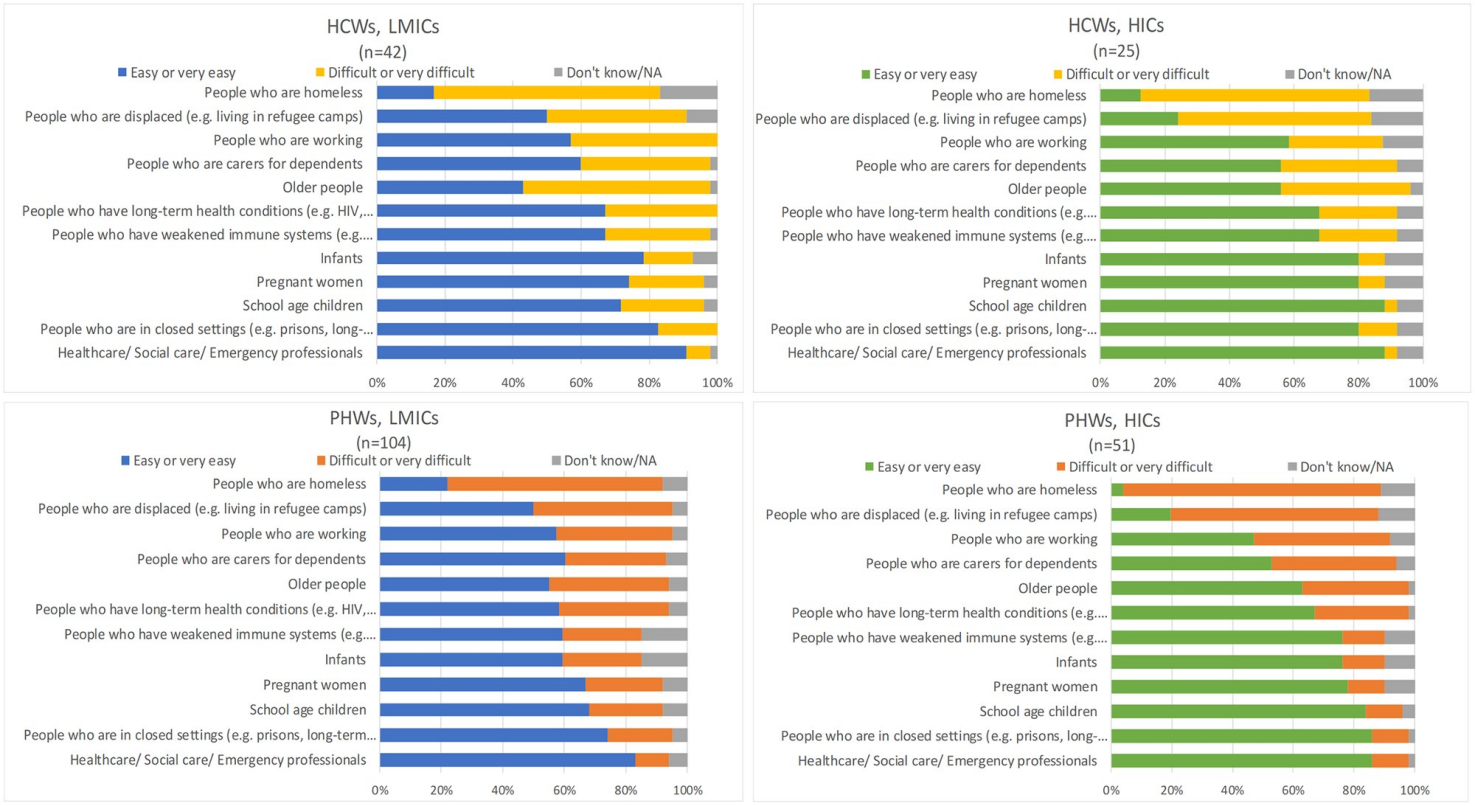
Public health workers’ and healthcare workers’ assessment of the practicality of implementing a COVID-19 vaccine for certain population groups.

Almost all respondents believed that a vaccine should be introduced to prevent COVID-19 (S9 Table in S1 File). Most indicated that specific population groups should be prioritised with people who have long-term health conditions and healthcare/social care/emergency professionals unanimously viewed as priority groups. Generally, across both income groups and professional groups, homeless people and those who are displaced were perceived as the most difficult groups to vaccinate (Fig 2).

The most commonly perceived barriers to implementing a COVID-19 vaccine were broadly similar across both professional and country income groups; public fear of vaccine safety and lack of vaccine supply were the main barriers (S11 Table in S1 File). PHWs in HICs and LMICs and HCWs in HICs also highlighted the fear of vaccine ineffectiveness, while lack of financial support and the vaccine being manufactured in another country were barriers reported by almost twice as many respondents in LMICs as in HICs (58% vs 33%). Qualitative data supported these findings: *“Already known or perceived NEO-COLONIAL interests that have been variously seen in these regards [PHW*, *LMIC]*. Furthermore, qualitative data suggested PHWs in both country income groups regarded misinformation as an additional barrier: *“Conspiracy theories on social media”* [PHW, LMIC]. Logistical challenges of vaccine distribution were also suggested: *“Lack of existing delivery systems outside of infancy and pregnancy*. *In LMICs there are no adult vaccination programmes—setting one up*, *building trust etc*. *would be hard” [PHW*, *LMIC]*.

## Discussion

This study provides evidence of the overall challenge of implementing COVID-19 interventions and highlights the nuances of contextual differences that influence the implementation of interventions and the associated barriers to implementation, as perceived by PHWs and HCWs in HICs and LMICs. Our results broadly illustrate little difference between HICs and LMICs in the perceived ease of implementation of a number of commonly reported NPIs, with the majority regarded as difficult to implement. However, some key differences between respondents in HICs and LMICs were noted in relation to implementing restrictions on schools and educational institutions and contact tracing methods, with these regarded as more challenging to implement in HICs than in LMICs. Lack of public support was the perceived overarching barrier to implementing NPIs among both professional and country income groups. On the other hand, important differences between the perceived barriers to implementing COVID-19 vaccination campaigns in LMICs and HICs were highlighted. In particular, a lack of financial support and the vaccine being manufactured in another country emerged as key perceived barriers in LMICs.

Our first findings indicate that travel restrictions were perceived as the most difficult type of intervention to implement generally, with domestic travel restrictions cited as the most difficult type of travel restriction to implement. Travel restrictions have been viewed as key mitigation measure during this pandemic and other outbreaks [42]. However, the health, economic and social consequences of travel restrictions are significant and our study suggests these are barriers to implementing travel restrictions. Furthermore, recent research suggests that the effectiveness of travel restrictions to limit the spread of disease is dependent on several factors, including levels of community transmission, travel volumes and other public health and social measures in place [43–45]. For example, it has been suggested that stringent travel restrictions may have minimal impact on local COVID-19 epidemics [43–45]. Yet, the majority of evidence is from modelling studies, and there is still a lack of real-world evidence to support this—an additional barrier indicated in our study to implementing travel restrictions. Moreover, COVID-19 variants of concern have the potential to cause increased transmissibility and pathogenicity or reduce vaccine efficacy [46]. Therefore, more research is required to understand the role of international travel and the spread of variants of concern.

Specifically, we found that restrictions on schools and educational institutions were perceived to be more difficult to implement in HICs than LMICs, with a lack of childcare support identified as the main barrier. This barrier was highlighted as a key issue relating to loss of income due to family members taking time off work to care for children, and this phenomenon has been identified in previous outbreak and pandemics in HICs [47, 48]. With evidence indicating the closing of schools and educational institutions as one of the most effective measures in slowing transmission [49], suggestions to overcome this barrier include valuing and accounting for care work in economic and social policies [50, 51].

Similarly, our data suggested that most contact tracing methods were perceived to be more difficult to implement in HICs than LMICs. A credible explanation for this is that LMICs are more familiar with outbreaks and have been drawing on lessons from previous experiences [52, 53]. Compared to LMICs, many HICs have less recent experience with large outbreaks of infectious disease and, despite being one of the most critical interventions for controlling outbreaks, many HICs have failed to implement effective contact tracing methods [52]. The perceived barriers to implementing contact tracing methods in HICs indicated in our study have come to the fore, including public distrust of health authorities and technologies, such as privacy concerns [52].

A lack of public support was the most commonly reported barrier to NPIs overall across both country income and professional groups. In contrast, evidence has suggested high public support for NPIs in most settings [21, 54]. However, one study highlighted that despite a high level of public support for COVID-19 measures, trust in government responses was considerably lower [54], as suggested by our qualitative data. Public trust is central to effective outbreak response strategies, and research has indicated that trust in COVID-19 interventions and perceptions of risk, differs between socioeconomic groups [38, 54]. Lower perception of risk is associated with lower adherence to public health interventions [37], as shown in our results. Potential explanations for higher perceptions of risk among HCWs and the general population include personal experience of disease or greater exposure to it [55]. Our study also indicated that perceptions of disease severity affect adherence to physical distancing, despite previous experience with other outbreaks. For example, people may not think they are at risk, as the effects of COVID-19 are not perceived to be immediately threatening compared to diseases like Ebola [56].

Furthermore, we found that trust and adherence to interventions differ by sociodemographic characteristics, which is supported by other work [36, 54, 57, 58]. Recognising these differences by sociodemographic groups can inform response strategies to improve trust and adherence to interventions. The apparent mismatch between PHWs’ and HCWs’ perception of lack of public support being a significant barrier to most interventions found in our study and other evidence suggesting significant public support for public health measures indicates that perhaps the public is willing to support interventions more than anticipated by PHWs and HCWs [21, 54]. Developing public trust is key to implementing interventions and ensuring adherence. Strategies include coherent and transparent communication, engaging communities when developing and implementing interventions and providing timely information that is user-friendly [59].

Our hypothetical findings on COVID-19 vaccination reflect the reality that has since transpired in several countries and contexts. For example, HCWs and PHWs across both country income groups prioritised similar population groups for COVID-19 vaccination, as policies implemented worldwide [60–62]. Furthermore, the key perceived barriers to implementing a vaccine—public fear of vaccine safety and lack of vaccine supply—have emerged worldwide [63, 64]. However, our study also highlighted differences between LMICs and HICs in perceptions of barriers to implementing a vaccine. PHWs and HCWs in LMICs perceived a lack of financial support and the vaccine being manufactured in another country as important barriers which have indeed materialised as the race to vaccinate the world continues [65]. Initiatives that attempt to address these barriers include COVAX (the vaccine initiative co-led by the Coalition for Epidemic Preparedness Innovations (CEPI), Gavi, the World Health Organization (WHO), and UNICEF) which aims to ensure fair and equitable access for COVID-19 vaccines in LMICs [66]. However, global health inequalities, including economic and geographical health disparities, have been further exposed as HICs accelerate ahead in vaccinating their populations, whilst the majority in LMICs remain unvaccinated [65]. Our qualitative data suggested that the legacy of colonialism in global health is seen as key barrier to implementing COVID-19 vaccines in LMICs, which further amplifies the need to decolonise global health if we are to overcome this pandemic as a global society [19, 21, 67, 68].

The strengths of employing survey methods using a snowball sampling technique to collect data include the ability to gather data rapidly and remotely from a variety of settings. However, we acknowledge there may be potential biases associated with the methods used in this study, such as, 1) response bias, including question order bias and low response rate through inadequate dissemination, limited accessibility, or lack of time in the context of a pandemic, and 2) sampling bias as a result of low response rate and self-selection of participants with people from the same demographic group. Thus, the findings of this study should be interpreted with caution. Nevertheless, this study provides a snapshot of the practicality of, and barriers to, implementation of COVID-19 interventions as perceived by PHWS and HCWs. We translated the survey into a limited number of languages and we received more responses mainly from the WHO Africa and Europe regions, in particular PHWs, thus we recognise these findings are not representative of all PHWs and HCWs. Furthermore, we recognise that the perceptions of PHWs and HCWs do not necessarily reflect community opinions or necessarily the reality of NPI and PI implementation and barriers, limiting the generalisability of the results.

This study provides initial evidence on PHW and HCW perceptions of COVID-19 interventions. PHWs and HCWs perceived considerable difficulty in implementing interventions particularly travel restrictions, across all contexts. Lack of public support was identified as a major barrier to implementing NPIs, highlighting the importance of community engagement and developing public trust in response strategies. HCWs and PHWs are viewed by the public as a critical source of public health information and have a wealth of knowledge about their local context. As a result, they should have a key role in informing the design of public health strategies. However, some important differences were noted between the perceptions in HICs and LMICs, including the barriers to implementing a COVID-19 vaccine. Thus, our study confirms there is no one-size-fits-all solution to implementing interventions. Future studies on this topic would benefit from larger sample sizes across different contexts.

## Supporting information

S1 File (PDF)

S1 Study survey (PDF)
