# Supplementary material for: A snapshot of the practicality and barriers to COVID-19 interventions: Public health and healthcare workers’ perceptions in high and low- and middle-income countries
Source: PLoS One. 2021 Nov 24;16(11):e0260041. doi: 10.1371/journal.pone.0260041 (PMC8612542; doi:10.1371/journal.pone.0260041)
Supplement: S1 File — (PDF) [file pone.0260041.s001.pdf]

Supporting Information:

A snapshot of the practicality and barriers to COVID-19 interventions: public health and healthcare workers' perceptions in high and low and middle-income countries.

Rosanna Glazik[1*], Hannah Moore[2], David Kennedy[3], Hilary Bower[4], Hana Rohan[5], Ashley Sharp[6], Anna C Seale[7]

[1] UK Public Health Rapid Support Team, Public Health England/London School of Hygiene & Tropical Medicine, London, United Kingdom [2] UK Field Epidemiology Training Programme (FETP), Public Health England, London, UK [3] UK Public Health Rapid Support Team, Public Health England/London School of Hygiene & Tropical Medicine, London, United Kingdom [4] UK Public Health Rapid Support Team, Public Health England/London School of Hygiene & Tropical Medicine, London, United Kingdom, [5] UK Public Health Rapid Support Team, Public Health England/London School of Hygiene & Tropical Medicine, London, United Kingdom [6] UK Public Health Rapid Support Team, Public Health England/London School of Hygiene & Tropical Medicine, London, United Kingdom [7] Department of Infectious Disease Epidemiology, London School of Hygiene & Tropical Medicine, London, United Kingdom.

Contents

S1 Table. Implementation of non-pharmaceutical interventions (NPIs) 3
S2 Table. Practicality of implementing non-pharmaceutical interventions (NPIs) 5
S3 Table. Barriers to implementing travel restrictions 7
S4 Table. Barriers to implementing restrictions on offices, businesses and institutions 9
S5 Table. Barriers to implementing restrictions on gatherings 11
S6 Table. Barriers to implementing individual measures 12
S7 Table. Barriers to implementing contact tracing methods 14
S8 Table. Barriers to implementing shielding measures for vulnerable populations 16
S9 Table. Vaccine implementation and prioritisation of population groups 18
S10 Table. Practicality of implementing a COVID-19 vaccine for certain population groups 19
S11 Table. Barriers to implementing a COVID-19 vaccine 21

S1 Table. Implementation of non-pharmaceutical interventions (NPIs)

Percentage of respondents reporting the following interventions implemented			
	**High-income countries n (%) N=51***	**Low and middle-income countries n (%) N=104†**	**P-value**
**Individual measures**	**51 (100)**	**101 (97)**	**0.551**
Face masks or face coverings	43 (84)	98 (94)	0.080
Handwashing	48 (94)	98 (94)	1.000
Physical distancing	48 (94)	84 (81)	-
Respiratory etiquette (e.g. covering mouth and nose with a tissue when coughing or sneezing)	42 (82)	75 (72)	0.233
Temperature monitoring	12 (24)	88 (85)	P<0.001
Other	2 (1)	3 (3)	-
Don't know	-	-	-
**Restrictions on gatherings of people**	**48 (94)**	**85 (82)**	**0.097**
Mass gatherings (e.g. conferences)	47 (92)	75 (72)	0.008
Private gatherings at home	35 (69)	23 (22)	p < 0.001
Private gatherings outside the home (e.g. funerals)	41 (80)	60 (58)	0.009
Public gatherings outside the home	46 (90)	66 (65	p < 0.001
Other	-	-	-
Don't know	-	-	-
**Restrictions on in-country (domestic) travel**	**27 (53)**	**33 (32)**	**0.018**
Curfew (restricted movement at a particular time)	5 (10)	23 (22)	0.099
Denial or restricted entry into other sub-national areas (e.g. states or provinces)	15 (29)	19 (18)	0.171
Non-essential movement restricted (e.g. stay at home order, lockdown)	14 (28)	26 (25)	0.894
Other	3 (6)	3 (3)	-
Suspended or restricted movement within the country (e.g. restricted domestic flights, public transport)	6 (12)	20 (19)	0.347
Don't know	-	-	-
**Restrictions on international travel**	**45 (88)**	**72 (69)**	**0.031**
Entry restrictions: denial of entry from specific countries or any country	19 (37)	32% (n=33)	0.615
Exit restrictions: denial of exit to specific countries	5 (10)	24% (n=25)	0.059
Suspended or restricted international flights	21 (41)	37% (n=38)	0.702
International land borders closure (partial or complete)	8 (26)	36 (35)	0.023
Visa restrictions	8 (16)	23 (22)	0.568
Entry screening and isolation or quarantine	39 (77)	50 (48)	0.001
Exit screening and isolation or quarantine	7 (14)	29 (28)	0.079
Suspended or restricted international ferries or ships	10 (20)	18 (17)	0.898
Other	-	-	-
Don't know	-	-	-

**Restrictions on offices, businesses and institutions**	**45 (88)**	**77 (74)**	**0.011**
Bars	42 (82)	50 (48)	p < 0.001
Conference centres	33 (65)	48 (46)	0.045
Cultural centres (e.g. museums, libraries)	28 (55)	43 (41)	0.155
Entertainment venues (e.g. cinemas)	37 (73)	56 (54)	0.040
Food shops/markets	24 (47)	29 (28)	0.029
Higher Education Institutions (e.g. universities)	25 (49)	65 (63)	0.154
Hotels	21 (41)	40 (39)	0.880
Offices	28 (55)	47 (45)	0.334
Places of worship	29 (57)	50 (48)	0.391
Restaurants	36 (71)	47 (45)	0.005
Schools (e.g. childcare centres, primary schools, secondary schools)	23 (45)	67 (64)	0.034
Sports facilities (e.g. clubs, fitness centres, gyms)	36 (71)	53 (51)	0.032
Other	-	-	-
Don’t know	-	-	-
**Contact tracing**	**49 (96)**	**88 (85)**	**0.107**
Physical contact tracing (e.g. conducted by people)	41 (80)	85 (82)	1.000
Mobile phone applications (apps) (e.g. Mobile phone global positioning system (GPS))	35 (74)	47 (45)	0.002
Closed-circuit television (CCTV)	-	3 (3)	-
Electronic tags	-	6 (6)	-
Bank card transactions	-	2 (2)	-
Other	-	3 (3)	-
Don't know	3 (6)	2 (2)	-

*Denominator is the total number of public health worker respondents in high-income countries

†Denominator is the total number of public health worker respondents in low and middle-income countries

.

S2 Table. Practicality of implementing non-pharmaceutical interventions (NPIs)

	High-income countries n (%) N=51*			Low and middle-income countries n (%) N=104†			P-value††
	Easy or very easy	Difficult or very difficult	Don't know	Easy or very easy	Difficult or very difficult	Don't know	
**Contact tracing methods**							
Closed-circuit television (CCTV)/ electronic tags as contact tracing methods	4 (8)	30 (59)	17 (33)	21 (20)	59 (57)	24 (23)	0.105
Contact tracing methods using peoples' bank card transactions	3 (6)	34 (67)	14 (27)	17 (16)	63 (61)	24 (23)	0.166
Mobile phone contact tracing apps/GPS-based tracing	15 (29)	31 (61)	5 (10)	40 (38)	58 (56)	6 (6)	0.422
Physical contact tracing methods (e.g. in-person visits, by phone)	8 (16)	43 (84)	-	31 (30)	70 (67)	3 (3)	0.063
**Travel restrictions**							
Denial or restricted entry into other sub-national areas (e.g. states or provinces)/suspended or restricted movement within the country (e.g. restricted domestic flights, public transport)	7 (14)	43 (84)	1 (2)	33 (32)	70 (67)	1 (1)	0.052
Entry/exit screening, isolation and quarantine measures in terms of practical challenges?	11 (22)	38 (75)	2 (4)	36 (35)	67 (64)	1 (1)	0.136
International entry/exit restrictions	11 (22)	39 (76)	1 (2)	30 (29)	72 (69)	2 (2)	0.626
Non-essential movement restrictions (e.g. stay at home order, lockdown)	9 (18)	42 (82)	-	21 (20)	82 (79)	1 (1)	0.720
Restrictions on international flights, ferries or ships	14 (27)	31 (61)	6 (12)	44 (42)	56 (64)	4 (4)	0.060
Visa restrictions (e.g. suspension or restrictions on work or travels visas)	28 (55)	14 (27)	9 (18)	58 (56)	41 (39)	5 (5)	0.022
**Individual measures**							
Face masks/ face coverings	23 (45)	27 (53)	1 (2)	47 (45)	56 (54)	1 (1)	0.874
Handwashing	35 (69)	15 (29)	1 (2)	61 (59)	42 (40)	1 (1)	0.380
Physical distancing	11 (22)	40 (78)	-	34 (33)	69 (66)	1 (1)	0.264
Respiratory etiquette (e.g. covering mouth and nose with a bent elbow or tissue coughing or sneezing)	34 (67)	16 (31)	1 (2)	47 (45)	56 (54)	1 (1)	0.030
Temperature monitoring	22 (43)	22 (43)	7 (14)	55 (53)	47 (45)	2 (2)	0.012
**Restrictions on businesses and institutions**							
Restrictions on bars, hotels and restaurants	14 (27)	36 (71)	1 (2)	48 (46)	52 (52)	2(2)	0.080
Restrictions on entertainment, conference, sport and cultural centres	21 (41)	27 (53)	3 (6)	60 (58)	42 (40)	2 (2)	0.097
Restrictions on food shops/markets	18 (35)	32 (63)	1 (2)	28 (27)	73 (70)	3 (3)	0.548
Restrictions on offices	22 (43)	28 (55)	1 (2)	49 (47)	53 (51)	2 (2)	0.896
Restrictions on places of worship	20 (39)	22 (43)	9 (18)	48 (46)	54 (52)	2 (2)	0.002
Restrictions on schools or higher education institutions	13 (25)	37 (73)	1 (2)	60 (58)	42 (40)	2 (2)	P < 0.001
**Restrictions on gatherings**							
Restrictions on mass gatherings (e.g. conferences)	39 (76)	11 (22)	1 (2)	61 (59)	41 (39)	2 (2)	0.085
Restrictions on private gatherings at home	4 (8)	46 (90)	1 (2)	26 (25)	74 (71)	4 (4)	0.015
Restrictions on private gatherings outside the home (e.g. funerals)	17 (33)	32 (63)	2 (4)	32 (31)	70 (67)	2 (2)	0.702
Restrictions on public gatherings outside the home	16 (31)	34 (67)	1 (2)	41 (39)	61 (59)	2 (2)	0.618
**Implementing shielding measures**							
Older people	12 (24)	32 (63)	7 (14)	32 (31)	62 (60)	10 (10)	0.545

People with long-term conditions or those with weakened immune systems	10 (20)	32 (63)	9 (18)	32 (31)	61 (59)	11 (11)	0.224
Pregnant women	13 (25)	27 (53)	11 (22)	32 (31)	60 (58)	12 (12)	0.249

*Denominator is the total number of public health worker respondents in high-income countries

†Denominator is the total number of public health worker respondents in low and middle-income countries

††P-value indicates difference between “Easy” or “Very easy” and “Difficult” or “Very difficult” categories only.

S3 Table. Barriers to implementing travel restrictions

	**High-income countries n (%) N=51***	**Low and middle- income countries n (%) N=104[†]**	**P-value**
**Barriers to implementing denial or restricted entry into other sub-national areas (e.g. states or provinces) /suspended or restricted movement within the country (e.g. restricted domestic flights, public transport)?**			
Lack of public support	38 (75)	55 (53)	0.016
Lack of enforcement	31 (61)	45 (43)	0.060
Lack of communication between policy-makers and the public	24 (47)	31 (30)	0.054
Lack of policies and guidelines	18 (35)	26 (25)	0.252
Lack of trained staff	15 (29)	19 (18)	0.171
Other	7 (14)	7 (7)	-
Don't know	-	1 (1)	-
**Barriers to implementing entry/exit screening, isolation and quarantine measures**			
Lack of enforcement	30 (59)	37 (36)	0.010
Lack of public support	26 (51)	45 (43)	0.463
Lack of financial support	22 (43)	32 (31)	0.181
Lack of trained staff	18 (35)	39 (38)	0.928
Lack of communication between policy-makers and the public	18 (35)	34 (33)	0.888
Lack of policies and guidelines	16 (31)	26 (25)	0.518
Lack of equipment	15 (29)	37 (36)	0.560
Other	6 (12)	5 (5)	-
Don't know	-	-	-
**Barriers to implementing international entry/exit restrictions**			
Lack of enforcement	30 (59)	44 (42)	0.078
Lack of communication between policy-makers and the public	26 (51)	41 (39)	0.233
Lack of public support	26 (51)	38 (37)	0.123
Lack of policies and guidelines	23 (45)	31 (30)	0.090
Lack of financial support	19 (37)	22 (21)	0.052
Lack of trained staff	16 (31)	27 (26)	0.606
Lack of equipment	7 (14)	27 (26)	0.128
Other	3 (6)	10 (10)	-
Don't know	-	-	-
**Barriers to implementing non-essential movement restrictions (e.g. stay at home order, lockdown)**			
Lack of public support	40 (78)	67 (64)	0.112
Lack of enforcement	33 (65)	42 (40)	0.007
Lack of communication between policy-makers and the public	28 (55)	41 (39)	0.099
Lack of financial support	17 (33)	36 (35)	1.000
Lack of policies and guidelines	20 (39)	26 (25)	0.102
Lack of trained staff	11 (22)	17 (16)	0.567
Other	7 (14)	14 (14)	-
Don't know	-	-	-
**Barriers to implementing restrictions on international flights, ferries or ships**			
Lack of public support	25 (49)	29 (28)	0.0157
Lack of communication between policy-makers and the public	21 (41)	36 (35)	0.536
Lack of enforcement	21 (41)	31 (32)	0.270
Lack of policies and guidelines	21 (41)	29 (28)	0.139
Lack of financial support	18 (35))	23 (22)	0.120
Lack of trained staff	13 (26)	26 (25)	1.000

Other	4 (8)	5 (5)	-
Don't know	-	-	-
**Barriers to implementing visa restrictions (e.g. suspension or restrictions on work or travels visas)**			
Lack of enforcement	6 (12)	18 (17)	0.509
Lack of financial support	4 (8)	12 (12)	0.582
Lack of policies and guidelines	8 (16)	21 (20)	0.658
Lack of public support	9 (18)	26 (25)	0.410
Lack of trained staff	3 (6)	10 (10)	0.547
Other	4 (8)	3 (3)	-
Don't know	-	2 (2)	-

*Denominator is the total number of public health worker respondents in high-income countries (including those who did not identify barriers).

†Denominator is the total number of public health worker respondents in low and middle-income countries (including those who did not identify barriers).

S4 Table. Barriers to implementing restrictions on offices, businesses and institutions

	High-income countries n (%) N=51*	Low and middle-income countries n (%) N=104†	P-value
**Barriers to implementing restrictions on bars, restaurants and hotels**			
Lack of public support	29 (57)	33 (32)	0.003
Lack of financial support for owners of bars, restaurants and hotels	26 (51)	40 (39)	0.151
Lack of enforcement	17 (33)	32 (31)	0.776
Lack of communication between policy-makers and the public	14 (28)	17 (16)	0.111
Lack of policies and guidelines	5 (10)	20 (19)	0.128
Other	2 (4)	2 (2)	-
Don’t know	-	-	-
**Barriers to implementing restrictions on entertainment, conference, sport and cultural centres**			
Lack of financial support for owners of restrictions on entertainment, conference, sport and cultural centres	22 (43)	30 (29)	0.112
Lack of public support	19 (37)	30 (29)	0.382
Lack of communication between policy-makers and the public	11 (22)	11 (11)	0.110
Lack of policies and guidelines	8 (16)	16 (15)	1.000
Lack of enforcement	8 (16)	16 (15)	1.000
Other	2 (4)	5 (5)	-
Don’t know	-	-	-
**Barriers to implementing restrictions on food shops/markets**			
Lack of public support	21 (41)	56 (54)	0.190
Lack of financial support for food shop/market owners	20 (39)	52 (50)	0.274
Lack of financial support for people who have been furloughed (put on temporary leave)	18 (35)	52 (50)	0.120
Lack of communication between policy-makers and the public	15 (29)	29 (28)	0.993
Lack of enforcement	16 (31)	34 (33)	1.000
Lack of policies and guidelines	10 (20)	23 (22)	0.881
Other	3 (6)	5 (5)	-
Don't know	1 (2)	-	-
**Barriers to implementing restrictions on offices**			
Lack of public support	21 (4)	30 (29)	0.175
Lack of financial support for people who have been furloughed (temporary leave)	18 (35)	37 (36)	1.000
Lack of support from employer to work at home	15 (29)	31 (30)	1.000
Lack of enforcement	17 (33)	27 (26)	0.443
Lack of communication between policy-makers and the public	14 (28)	19 (18)	0.270
Lack of policies and guidelines	10 (20)	22 (21)	1.000
Other	3 (6)	4 (4)	-
Don’t know	-	-	-
**Barriers to implementing restrictions on places of worship**			
Lack of public support	13 (26)	42 (40)	0.101
Lack of enforcement	14 (28)	23 (22)	0.595
Lack of communication between policy-makers and the public	8 (16)	26 (25)	0.267
Lack of policies and guidelines	7 (14)	22 (21)	0.371
Other	2 (4)	3 (3)	-
Don’t know	-	-	-

**Barriers to implementing restrictions on schools or higher education institutes**			
Lack of childcare support	31 (61)	29 (28)	P < 0.001
Lack of remote/distance learning support (e.g. limited or no access to remote education resources, laptops, internet)	27 (53)	32 (31)	0.013
Lack of public support	25 (49)	21 (20)	P < 0.001
Lack of communication between policy-makers and the public	20 (39)	18 (17)	0.005
Lack of enforcement	15 (29)	16 (15)	0.066
Lack of policies and guidelines	13 (26)	19 (18)	0.405
Other	7 (14)	4 (4)	-
Don't know	-	-	-

*Denominator is the total number of public health worker respondents in high-income countries (including those who did not identify barriers).

[†]Denominator is the total number of public health worker respondents in low and middle-income countries (including those who did not identify barriers).

S5 Table. Barriers to implementing restrictions on gatherings

	High-income countries n (%) N=51*	Low and middle-income countries n (%) N=104†	P-value
**Barriers to implementing restrictions on mass gatherings (e.g. conferences)**			
Lack of public support	7 (14)	28 (27)	0.101
Lack of enforcement	7 (14)	27 (26)	0.128
Lack of resources to support mass gathering remotely (e.g. technology infrastructure)	7 (14)	25 (24)	0.201
Socio-cultural practices and beliefs	6 (12)	26 (25)	0.089
Lack of communication between policy-makers and the public	3 (6)	15 (14)	0.181
Lack of policies and guidelines	2 (4)	16 (15)	0.059
Other	1 (2)	2 (2)	-
Don’t know	-	-	-
**Barriers to implementing restrictions on private gatherings at home**			
Lack of public support	42 (82)	60 (58)	0.004
Lack of enforcement	34 (67)	45 (43)	0.010
Lack of communication between policy-makers and the public	19 (37)	24 (23)	0.097
Lack of policies and guidelines	7 (14)	20 (19)	0.533
Other	5 (10)	5 (5)	-
Don't know	-	1 (1)	-
**Barriers to implementing restrictions on private gatherings outside the home (e.g. funerals)**			
Lack of public support	29 (57)	63 (61)	0.788
Lack of enforcement	19 (37)	45 (43)	0.589
Lack of communication between policy-makers and the public	16 (31)	30 (29)	0.892
Lack of policies and guidelines	6 (12)	18 (17)	0.509
Other	1 (2)	5 (5)	-
Don't know	-	-	-
**Barriers to implementing restrictions on public gatherings outside the home**			
Lack of public support	31 (61)	54 (52)	0.384
Lack of enforcement	22 (43)	40 (39)	0.701
Socio-cultural practices and beliefs	21 (41)	41 (39)	0.972
Lack of communication between policy-makers and the public	20 (39)	22 (21)	0.029
Lack of policies and guidelines	7 (14)	23 (22)	0.305
Other	4 (8)	2 (2)	-
Don't know	-	-	-

*Denominator is the total number of public health worker respondents in high-income countries (including those who did not identify barriers).

†Denominator is the total number of public health worker respondents in low and middle-income countries (including those who did not identify barriers).

S6 Table. Barriers to implementing individual measures

	High-income countries n (%) N=51*	Low and middle-income countries n (%) N=104†	P-value
**Barriers to implementing face masks/ face coverings**			
Lack of public support	17 (33)	37 (36)	0.923
Misinformation (e.g. rumours or myths)	16 (31)	36 (35)	0.825
Socio-cultural beliefs and practices	11 (22)	33 (32)	0.259
Lack of enforcement	12 (24)	27 (26)	0.896
Social stigma	7 (14)	19 (18)	0.629
Lack of communication between policy-makers and the public	7 (14)	14 (14)	1.000
Lack of resources (e.g. face masks/ face coverings)	6 (12)	39 (38)	0.002
Lack of communication between healthcare providers and the public	5 (10)	16 (15)	0.481
Lack of policies and guidelines	4 (8)	10 (10)	1.000
Other	6 (12)	3 (3)	-
Don't know	-	1 (1)	-
**Barriers to implementing handwashing**			
Lack of resources (e.g. handwashing areas, water, soap, alcohol rub)	10 (20)	34 (33)	0.132
Lack of enforcement	8 (16)	19 (18)	0.863
Lack of public support	4 (8)	24 (23)	0.025
Misinformation (e.g. rumours or myths)	4 (8)	23 (22)	0.041
Socio-cultural beliefs and practices	6 (12)	15 (14)	0.838
Lack of communication between healthcare providers and the public	2 (4)	16 (15)	0.059
Lack of communication between policy-makers and the public	2 (4)	11 (11)	0.223
Social stigma	1 (2)	10 (10)	0.103
Lack of policies and guidelines	1 (2)	7 (7)	0.273
Other	2 (4)	4 (4)	-
Don't know	-	-	-
**Barriers to implementing physical distancing**			
Not possible to maintain physical distance at workplace due to the nature of the work	23 (45)	49 (47)	0.948
Lack of public support	22 (43)	55 (53)	0.332
Socio-cultural beliefs and practices	19 (37)	42 (40)	0.842
Lack of enforcement	20 (39)	38 (37)	0.883
Misinformation (e.g. rumours or myths)	19 (37)	29 (28)	0.317
Lack of communication between healthcare providers and the public	8 (16)	20 (19)	0.751
Lack of communication between policy-makers and the public	11 (22)	18 (17)	0.675
Social stigma	3 (6)	18 (17)	0.078
Lack of policies and guidelines	4 (8)	11 (11)	0.775
Other	8 (16)	4 (4)	-
Don't know	-	-	-
**Barriers to implementing respiratory etiquette (e.g. covering mouth and nose with a bent elbow or tissue when coughing or sneezing)**			
Lack of public support	5 (10)	35 (34)	0.003
Socio-cultural beliefs and practices	9 (18)	28 (27)	0.284
Misinformation (e.g. rumours or myths)	6 (12)	30 (29)	0.030
Social stigma	1 (2)	20 (19)	0.002
Lack of communication between healthcare providers and the public	3 (6)	18 (17)	0.078
Lack of enforcement	5 (10)	14 (14)	0.695
Lack of communication between policy-makers and the public	1 (2)	12 (12)	0.062
Lack of policies and guidelines	-	10 (10)	0.052
Other	2 (4)	4 (4)	-
Don't know	-	-	-

**Barriers to implementing temperature monitoring**			
Lack of equipment	14 (28)	39 (38)	0.290
Lack of enforcement	10 (20)	18 (17)	0.898
Misinformation (e.g. rumours or myths)	9 (18)	18 (17)	1.000
Lack of public support	6 (12)	22 (21)	0.228
Lack of policies and guidelines	7 (14)	8 (8)	0.366
Socio-cultural beliefs and practices	5 (10)	13 (13)	0.822
Lack of communication between healthcare providers and the public	3 (6)	9 (9)	0.752
Lack of communication between policy-makers and the public	4 (8)	8 (8)	1.000
Social stigma	2 (4)	10 (10)	0.339
Other	7 (14)	3 (3)	-
Don't know	-	-	-

*Denominator is the total number of public health worker respondents in high-income countries (including those who did not identify barriers).

†Denominator is the total number of public health worker respondents in low and middle-income countries (including those who did not identify barriers).

S7 Table. Barriers to implementing contact tracing methods

	High-income countries n (%) N=51*	Low and middle-income countries n (%) N=104†	P-value
**Barriers to implementing closed-circuit television (CCTV)/ electronic tags**			
Privacy laws	24 (47)	17 (16)	P < 0.001
Lack of public support	19 (47)	23 (22)	0.072
Lack of technology infrastructure	12 (24)	47 (45)	0.015
Social stigma	9 (18)	8 (8)	0.112
Socio-cultural beliefs and practices	8 (16)	11 (11)	0.515
Lack of trained staff	7 (14)	24 (23)	0.249
Misinformation (e.g. rumours or myths)	7 (14)	8 (8)	0.366
Lack of policies and guidelines	6 (12)	19 (18)	0.422
Lack of communication between policy-makers and the public	4 (8)	7 (7)	0.752
Lack of enforcement	4 (8)	15 (14)	0.304
Lack of communication between healthcare providers and the public	2 (4)	10 (10)	0.339
Other	1 (2)	2 (2)	-
Don't know	-	-	-
**Barriers to implementing contact tracing methods using peoples' bank card transactions**			
Privacy laws	30 (59)	23 (22)	P < 0.001
Lack of public support	19 (37)	23 (22)	0.072
Lack of technology infrastructure	10 (20)	44 (42)	0.009
Lack of policies and guidelines	8 (16)	21 (20)	0.648
Misinformation (e.g. rumours or myths)	8 (16)	11 (11)	0.515
Lack of trained staff	7 (14)	21 (20)	0.447
Socio-cultural beliefs and practices	7 (14)	13 (13)	1.000
Social stigma	5 (10	8 (8)	0.891
Lack of enforcement	2 (4)	14 (14)	0.091
Lack of communication between policy-makers and the public	4 (8)	10 (10)	1.000
Lack of communication between healthcare providers and the public	3 (6)	6 (9)	0.752
Other	2 (4)	5 (5)	-
Don't know	-	1 (1)	-
**Barriers to implementing mobile phone contact tracing apps / GPS-based tracing**			
Lack of technology infrastructure	17 (33)	42 (40)	0.501
Privacy laws	17 (33)	17 (16)	0.028
Lack of public support	14 (28)	25 (24)	0.793
Misinformation (e.g. rumours or myths)	13 (26)	15 (14)	0.144
Lack of communication between policy-makers and the public	11 (22)	11 (11)	0.110
Lack of trained staff	9 (18)	26 (25)	0.410
Lack of enforcement	6 (12)	15 (14)	0.838
Socio-cultural beliefs and practices	7 (14)	17 (16)	0.851
Lack of communication between healthcare providers and the public	7 (14)	11 (11)	0.758
Social stigma	4 (8)	12(12)	0.582
Lack of policies and guidelines	3 (6)	15 (14)	0.181
Other	6 (12)	5 (5)	-
Don't know	-	1 (1)	-

**Barriers to implementing physical contact tracing methods (e.g. in-person visits, by phone)**			
Lack of trained staff	34 (67)	44 (42)	0.007
Lack of public support	26 (51)	44 (42)	0.400
Lack of technology infrastructure	22 (43)	45 (43)	1.000
Misinformation (e.g. rumours or myths)	20 (39)	29 (28)	0.214
Lack of communication between policy-makers and the public	20 (39)	13 (13)	P < 0.001
Privacy laws	18 (35)	19 (18)	0.033
Lack of enforcement	16 (31)	24 (23)	0.361
Social stigma	11 (22)	36 (35)	0.140
Socio-cultural beliefs and practices	12 (24)	30 (29)	0.612
Lack of communication between healthcare providers and the public	11 (22)	20 (19)	0.898
Lack of policies and guidelines	5 (10)	14 (14)	0.695
Other	7 (14)	7 (7)	-
Don't know	-	1 (1)	-

*Denominator is the total number of public health worker respondents in high-income countries (including those who did not identify barriers).

†Denominator is the total number of public health worker respondents in low and middle-income countries (including those who did not identify barriers).

S8 Table. Barriers to implementing shielding measures for vulnerable populations

	Public Health Workers (PHWs)			Healthcare Workers (HCWs)		
	**High-income countries n (%) N=51***	**Low and middle-income countries n (%) N=104†**	**P-value**	**High-income countries n (%) N=25††**	**Low and middle- income countries n (%) N=42¶**	**P-value**
**Older people**						
Lack of home support for older people (e.g. medical care, supply of food/medications)	24 (47)	54 (52)	0.691	11 (44)	26 (62)	0.241
Other	13 (26)	4 (4)	-	6 (24)	14 (33)	-
Lack of public support	13 (26)	32 (31)	0.623	4 (16)	14 (33)	0.595
Socio-cultural beliefs and practices	10 (20)	34 (33)	0.132	4 (16)	13 (31)	0.248
Misinformation (e.g. rumours or myths)	8 (16)	21 (20)	0.648	5 (20)	2 (5)	0.027
Lack of enforcement	8 (16)	19 (18)	0.863	2 (8)	11 (26)	0.186
Lack of communication between healthcare providers and the public	8 (16)	16 (15)	1.000	3 (12)	16 (38)	0.109
Lack of communication between policy-makers and the public	6 (12)	13 (13)	1.000	3 (12)	14 (33)	0.159
Lack of policies and guidelines	5 (10)	22 (21)	0.127	3 (12)	10 (24)	0.081
Social stigma	3 (6)	17 (16)	0.078	2 (8)	10 (24)	0.342
Don't know	-	-	-	-	-	-
**People with long-term conditions or those with weakened immune systems**						
Lack of home support for people with long-term conditions or those with weakened immune systems (e.g. medical care, supply of food/medications)	24 (47)	44 (42)	0.698	8 (32)	24 (57)	0.081
Other	14 (28)	2 (2)	-	7 (28)	11 (26)	-
Lack of public support	10 (20)	30 (29)	0.298	3 (12)	16 (38)	0.027
Lack of communication between healthcare providers and the public	8 (16)	21 (20)	0.648	4 (16)	13 (31)	0.023
Lack of communication between policy-makers and healthcare providers	8 (16)	12 (12)	0.639	1 (4)	16 (38)	0.190
Lack of communication between policy-makers and the public	6 (12)	13 (13)	1.000	1 (4)	13 (31)	1.000
Socio-cultural beliefs and practices	6 (12)	22 (21)	0.228	1 (4)	12 (29)	0.011
Lack of enforcement	5 (10)	21 (20)	0.162	1 (4)	12 (29)	0.02
Lack of policies and guidelines	5 (10)	21 (20)	0.162	1 (4)	13 (31)	0.2
Misinformation (e.g. rumours or myths)	4 (8)	16 (15)	0.214	2 (8)	10 (24)	0.001
Social stigma	4 (8)	19 (18)	0.098	2 (8)	1 (2)	0.011
Don't know	-	-	-	-	-	-
**Pregnant women**						
Lack of financial support for pregnant women	20 (39)	41 (39)	1.000	10 (40)	19 (45)	0.870
Lack of home support for pregnant women (e.g. medical care, supply of food/medications)	15 (29)	40 (39)	0.353	9 (36)	21 (50)	0.389
Lack of public support	11 (22)	27 (26)	0.690	8 (32)	15 (36)	0.047
Other	9 (18)	2 (2)	-	8 (32)	14 (44)	-
Socio-cultural beliefs and practices	6 (12)	27 (26)	0.068	6 (24)	15 (36)	0.489
Lack of communication between healthcare providers and the public	6 (12)	17 (16	0.608	5 (20)	13 (31)	0.022
Lack of communication between policy-makers and the public	6 (12)	14 (14)	0.967	3 (12)	15 (36)	0.965
Lack of enforcement	5 (10)	18 (17)	0.320	2 (8)	12 (29)	0.063

Misinformation (e.g. rumours or myths)	5 (10)	15 (14)	0.581	-	12 (29)	0.467
Lack of policies and guidelines	4 (8)	22 (21)	0.041	-	10 (24)	1.000
Social stigma	3 (6)	14 (14)	0.183	4 (16)	1 (2)	0.009
Don't know	-	1 (1)	-	-	-	-

*Denominator is the total number of public health worker respondents in high-income countries (including those who did not identify barriers).

†Denominator is the total number of public health worker respondents in low and middle-income countries (including those who did not identify barriers).

††Denominator is the total number of healthcare worker respondents in high-income countries (including those who did not identify barriers).

¶ Denominator is the total number of healthcare worker respondents in low and middle-income countries (including those who did not identify barriers).

S9 Table. Vaccine implementation and prioritisation of population groups

**Do you think a vaccine, if developed, should be introduced to prevent COVID-19?**						
	**Healthcare workers (HCWs)**			**Public health workers (PHWs)**		
	**High-income countries n (%) N=25***	**Low and middle-income countries n (%) N=42†**	**P-value**	**High-income countries n (%) N=51††**	**Low and middle-income countries n (%) N=104¶**	**P-value**
Yes	22 (88)	41 (98)		50 (98)	94 (90)	
No	2 (8)	-	0.255	1 (2)	7 (7)	0.261
Don't know	1 (4)	1 (2)	-	-	3 (3)	-
**Do you think a vaccine should be prioritised for certain population groups (e.g. pregnant women, healthcare workers, older people)?**						
Yes	13 (52)	24 (57)		40 (78)	50 (48)	
No – everyone should be vaccinated	9 (36)	17 (40)	1.000	10 (20)	41 (39)	0.005
NA	3 (12)	1 (2)	-	1 (2)	10 (10)	-
Don't know			-	-	3 (3)	-
**Which of the following population groups do you think a vaccine should be prioritised for? (please select up to 3 groups)**						
Healthcare/ Social care/ Emergency professionals	12 (48)	10 (24)	0.076	24 (47)	22 (21)	0.002
Infants	-	1 (2)	-	3 (6)	8 (8)	0.099
Older people	9 (36)	9 (2)	0.309	20 (40)	23 (22)	0.041
People who are carers for dependents	1 (4)	1 (2)	1.000	2 (4)	4 (4)	1.000
People who are displaced (e.g. living in refugee camps)	2 (8)	1 (2)	0.551	1 (2)	2 (2)	1.000
People who are in closed settings (e.g. prisons, long-term care facilities)	2 (8)	2 (5)	0.626	11 (22)	7 (7)	0.015
People who are working	-	2 (5)	-	1 (2)	-	-
People who have long-term health conditions (e.g. diabetes, heart disease)	8 (32)	15 (36)	0.965	24 (27)	25 (24)	0.007
People who have weakened immune systems (e.g. cancer or transplant patients)	5 (20	9 (21)	1.000	22 (43)	20 (19)	0.003
Pregnant women	-	9 (21)	-	1 (2)	19 (18)	0.004
Homeless people	-	-	-	1 (2)	1 (1)	0.551
School age children	-	3 (7)	-	3 (6)	7 (7)	1.000
Don't know	-	-	-	1 (2)	-	-
Other	-	-	-	1 (2)	1 (1)	-

*Denominator is the total number of healthcare worker respondents in high-income countries.
†Denominator is the total number of healthcare worker respondents in low and middle-income countries.
††Denominator is the total number of public health worker respondents in high-income countries.
¶ Denominator is the total number of public health worker respondents in low and middle-income countries.

S10. Practicality of implementing a COVID-19 vaccine for certain population groups

**How easy do you think it is to provide a COVID-19 vaccine to these population groups in terms of practical challenges?**						
	**Public health workers (PCWs)**			**Healthcare workers (HCWs)**		
	**High-income countries n (%) N=51***	**Low and middle-income countries n (%) N=104[†]**	**P-value***	**High-income countries n (%) N=25[††]**	**Low and middle-income countries n (%) N=42[¶]**	**P-value[§]**
**Healthcare/ Social care/ Emergency professionals**						
Easy or very easy	44 (86)	87 (84)		22 (88)	38 (91)	
Difficult or very difficult	6 (12)	11 (11)	1.000	1 (4)	3 (7)	1.000
Don't know	-	5 (5)		-	-	
Not applicable (NA)	1 (2)	1 (1)		2 (8)	1 (2)	
**People who are in closed settings (e.g. prisons, long-term care facilities)**						
Easy or very easy	4 (86)	77 (74)		20 (80)	34 (81)	
Difficult or very difficult	6 (12)	22 (21)	0.198	3 (12)	7 (17)	1.000
Don't know	-	5 (5)		-		
Not applicable (NA)	1 (2)	-		2 (8)	-	
**School age children**						
Easy or very easy	43 (84)	71 (68)		22 (88)	30 (71)	
Difficult or very difficult	6 (12)	25 (24)	0.089	1 (4)	10 (24)	0.044
Don't know	1 (2)	5 (5)		-	1 (2)	
Not applicable (NA)	1 (2)	3 (3)		2 (8)	1 (2)	
**Pregnant women**						
Easy or very easy	40 (78)	70 (67)		20 (80)	31 (74)	
Difficult or very difficult	6 (12)	26 (25)	0.097	2 (8)	9 (22)	0.300
Don't know	2 (4)	6 (6)	-	1 (4)	1 (2)	
Not applicable (NA)	3 (6)	2 (2)	-	2 (8)	1 (2)	
**Infants**						
Easy or very easy	39 (77)	62 (60)		20 (80)	32 (76)	
Difficult or very difficult	7 (14)	27 (26)	0.087	2 (8)	6 (14)	0.697
Don't know	2 (40	5 (5)	-	-	1 (2)	
Not applicable (NA)	3 (6)	10 (10)	-	3 (12)	2 (5)	
**People who have weakened immune systems (e.g. tuberculosis, cancer or transplant patients)**						
Easy or very easy	39 (77)	62 (60)		17 (68)	28 (67)	
Difficult or very difficult	7 (14)	27 (26)	0.122	6 (24)	13 (31)	0.852
Don't know	2 (4)	5 (5)	-	1 (4)	-	
Not applicable (NA)	3 (6)	10 (10)	-	1 (4)	1 (2)	
**People who have long-term health conditions (e.g. HIV, diabetes, heart disease)**						
Easy or very easy	34 (67)	61 (59)		17 (68)	28 (67)	
Difficult or very difficult	16 (31)	37 (36)	0.610	6 (24)	14 (33)	0.746
Don't know	1 (2)	6 (6)	-	1 (4)	-	
Not applicable (NA)	-	-	-	1 (4)	-	
**Older people**						
Easy or very easy	32 (63)	57 (55)		14 (56)	18 (43)	
Difficult or very difficult	18 (35)	41 (49)	0.611	10 (40)	23 (55)	0.386
Don't know	1 (2)	6 (6)	-	-	-	
Not applicable (NA)	-	-	-	1 (4)	1 (2)	
**People who are carers for dependents**						
Easy or very easy	27 (53)	63 (61)		14 (56)	25 (60)	
Difficult or very difficult	21 (41)	34 (33)	0.404	9 (36)	16 (38)	1.000
Don't know	2 (4)	6 (6)		-	-	
Not applicable (NA)	1 (2)	1 (1)		2 (8)	1 (2)	
**People who are working**						
Easy or very easy	24 (47)	60 (58)		14 (56)	24 (57)	
Difficult or very difficult	23 (45)	39 (38)	0.362	7 (28)	18 (43)	0.649
Don't know	2 (4)	4 (4)		1 (4)	-	
Not applicable (NA)	2 (4)	1 (1)		2 (8)	-	
**People who are displaced (e.g. living in refugee camps)**						
Easy or very easy	10 (20)	52 (50)		6 (24)	21 (50)	
Difficult or very difficult	35 (69)	47 (45)	0.001	15 (60)	17 (41)	0.090
Don't know	4 (8)	5 (5)		2 (8)	1 (2)	
Not applicable (NA)	2 (4)	-		2 (8)	3 (7)	
**People who are homeless**						

Easy or very easy	2 (4)	23 (22)	0.010	3 (12)	7 (17)	0.731
Difficult or very difficult	43 (84)	73 (70)		17 (68)	28 (67)	
Don't know	5 (10)	5 (5)		2 (8)	3 (7)	
Not applicable (NA)	1 (2)	3 (3)		2 (8)	4(10)	

*Denominator is the total number of public health worker respondents in high-income countries.

†Denominator is the total number of public health worker respondents in low and middle-income countries.

††Denominator is the total number of healthcare worker respondents in high-income countries.

¶Denominator is the total number of healthcare worker respondents in low and middle-income countries.

§P-value indicates difference between “Easy” or “Very easy” and “Difficult” or “Very difficult” categories only.

S11 Table. Barriers to implementing a COVID-19 vaccine

	Public Health Workers (PHWs)			Healthcare workers (HCWs)		
	**High-income countries n (%) N=51***	**Low and middle-income countries n (%) N=104†**	**P-value***	**High-income countries n (%) N=25††**	**Low and middle-income countries n (%) N=42¶**	**P-value**
Public fear of vaccine safety	46 (90)	81 (78)	0.099	18 (72)	28 (67)	0.134
Lack of vaccine supply	39 (77)	80 (77)	1.000	16 (64)	35 (83)	0.855
Public fear of vaccine effectiveness	38 (75)	66 (64)	0.233	15 (60)	18 (43)	0.269
Lack of public support	26 (51)	50 (48)	0.866	9 (36)	24 (57)	0.703
Lack of communication between policy-makers and the public	24 (47)	38 (37)	0.279	9 (36)	15 (36)	1.000
Socio-cultural beliefs and practices	22 (43)	60 (58)	0.125	9 (36)	11 (26)	0.155
Lack of financial support	20 (39)	65 (63)	0.010	7 (28)	15 (36)	0.002
Lack of communication between healthcare providers and the public	17 (33)	42 (40)	0.501	5 (20)	26 (62)	0.567
Lack of equipment (e.g. cold chain)	17 (33)	49 (47)	0.145	8 (32)	18 (43)	0.533
Lack of trained staff	13 (26)	34 (33)	0.465	5 (20)	22 (52)	0.024
Lack of policies and guidelines	12 (24)	35 (34)	0.270	4 (16)	16 (38)	0.962
Vaccine is manufactured in another country	10 (20)	53 (51)	P < 0.001	1 (4)	11 (26)	0.018
Other	6 (12)	4 (4)	-	1 (4)	6 (14)	-
Social stigma	5 (10)	28 (27)	0.025	2 (8)	1 (2)	0.244
Don't know	-	2 (2)	-	-	1 (2)	-

*Denominator is the total number of public health worker respondents in high-income countries.

†Denominator is the total number of public health worker respondents in low and middle-income countries.

††Denominator is the total number of healthcare worker respondents in high-income countries.

¶ Denominator is the total number of healthcare worker respondents in low and middle-income countries.
