# Supplementary material for: A snapshot of the practicality and barriers to COVID-19 interventions: Public health and healthcare workers’ perceptions in high and low- and middle-income countries
Source: PLoS One. 2021 Nov 24;16(11):e0260041. doi: 10.1371/journal.pone.0260041 (PMC8612542; doi:10.1371/journal.pone.0260041)
Supplement: S1 Study survey — (PDF) [file pone.0260041.s002.pdf]

COVID-19 Interventions Survey (Healthcare Professionals)

Page 1: Introduction

This survey on public health interventions to respond to COVID-19 is being carried out by researchers from the **UK Public Health Rapid Support Team (UK-PHRST).**

COVID-19 is a challenge worldwide and changing everyone's lives. Public health interventions with restrictions on a scale not seen in modern times are in place; at the same time new methods to support containment are being developed, medications are being tested and in the longer term vaccines may prevent the disease.

This survey aims to gain understanding of public health and healthcare workers' professional assessment of current and future COVID-19 interventions, specifically the practicality of, and barriers to, implementation in their context.

Your participation is important **to share learning to inform the COVID-19 response in your country and in others**. We aim to disseminate the learning from this survey through academic and online platforms to share professional experience worldwide in response to the pandemic.

About This Survey

- This research has received approval from the London School of Hygiene and Tropical Medicine (LSHTM) Ethics Committee.
- The study is for healthcare and public health professionals worldwide.
- This study is anonymous, which means that we will not know who you are, and no information that could indentify you will be used in any report or publication.
- The survey will take around 20 minutes to complete.
- The line at the top of the screen shows your progress through the survey.
- Data will be stored securely at LSHTM's repository.
- This research will comply with the EU General Data Protection Regulation (GDPR) for research data management and storage.
- Summarised results will be shared through the UK-PHRST Knowledge hub and in publications.

We are hoping to get as many healthcare and public health workers worldwide to complete this survey, so please share widely.

For more information please contact uk-phrst@lshtm.ac.uk.

Thank you for your time and help.

1. Are you a **healthcare professional** aged **over 18 years** and **do you agree** to take part in this survey?

- ○ Yes
- ○ No

Page 2: Information about survey questions

Please answer the following questions based on your **professional** opinion with respect to the **country you are most associated with (e.g. where you work or where you live) at the time you complete the survey.**

Page 3: Shielding

Shielding is a measure to protect vulnerable people from coming into contact with COVID-19, by restricting interactions between them and others.

2. Are any population groups being shielded?

- Yes
- No
- Don't know

2.a. Which population groups are being shielded?

- Pregnant women
- Older people
- People who have long-term health conditions (e.g. HIV, diabetes, heart disease)
- People who have weakened immune systems (e.g. tuberculosis (TB), cancer or transplant patients)
- Don't know
- Other

2.a.i. If you selected Other, please specify:

2.b. How easy do you think it is to implement shielding measures for pregnant women in terms of practical challenges? *
Required

- Very easy
- Easy
- Difficult
- Very difficult
- Don't know

2.b.i. Which of the following do you think are barriers to implementing shielding measures for pregnant women?

- Lack of enforcement
- Lack of policies and guidelines
- Lack of communication between policy-makers and the public
- Lack of communication between healthcare providers and the public
- Lack of public support
- Lack of home support for pregnant women (e.g. medical care, supply of food/medications)
- Lack of financial support for pregnant women
- Social stigma

- Misinformation (e.g. rumours or myths)
- Socio-cultural beliefs and practices
- Other
- Don't know

2.b.i.a. If you selected Other, please specify:

2.c. How easy do you think it is to implement shielding measures for older people in terms of practical challenges?

- Very easy
- Easy
- Difficult
- Very difficult
- Don't know

2.c.i. Which of the following do you think are barriers to implementing shielding measures for older people?

- Lack of enforcement
- Lack of policies and guidelines
- Lack of communication between policy-makers and the public
- Lack of communication between healthcare providers and the public
- Lack of public support
- Lack of home support for older people (e.g. medical care, supply of food/medications)
- Social stigma
- Misinformation (e.g. rumours or myths)
- Socio-cultural beliefs and practices
- Other
- Don't know

2.c.i.a. If you selected Other, please specify:

2.d. How easy do you think it is to implement shielding measures for people with long-term conditions or those with weakened immune systems in terms of practical challenges?

- Very easy
- Easy
- Difficult

- Very difficult
- Don't know

2.d.i. Which of the following do you think are barriers to implementing shielding measures for people with long-term conditions or those with weakened immune systems?

- Lack of enforcement
- Lack of policies and guidelines
- Lack of communication between policy-makers and the public
- Lack of communication between policy-makers and healthcare providers
- Lack of communication between healthcare providers and the public
- Lack of public support
- Lack of home support for people with long-term conditions or those with weakened immune systems (e.g. medical care, supply of food/medications)
- Social stigma
- Misinformation (e.g. rumours or myths)
- Socio-cultural beliefs and practices
- Other
- Don't know

2.d.i.a. If you selected Other, please specify:

Page 4: Vaccines

The following questions refer to **vaccine use**, assuming a safe and effective vaccine for COVID-19 can be developed.

3. Do you think a vaccine, if developed, should be introduced to prevent COVID-19?

- Yes
- No
- Don't know

3.a. Do you think a vaccine should be prioritised for certain popualtion groups (e.g. pregnant women, healthcare workers, older people)?

- Yes
- No – everyone should be vaccinated
- Don't know

3.a.i. Which of the following population groups do you think a vaccine should be prioritised for? (please select up to 3 groups)

- Infants
- School age children
- Pregnant women
- People who are working
- People who are carers for dependents
- Older people
- People who have long-term health conditions (e.g. diabetes, heart disease)
- People who have weakened immune systems (e.g. cancer or transplant patients)
- People who are in closed settings (e.g. prisons, long-term care facilities)
- People who are displaced (e.g. living in refugee camps)
- Healthcare/ Social care/ Emergency professionals
- Other

3.a.i.a. If you selected Other, please specify:

3.b. How easy do you think it is to provide a COVID-19 vaccine to these population groups in terms of practical challenges?

	Very easy	Easy	Difficult	Very difficult	Don't know	Not applicable (NA)
Infants						
School age children						
Pregnant women						

People who are working	☐	☐	☐	☐	☐	☐
People who are carers for dependents	☐	☐	☐	☐	☐	☐
Older people	☐	☐	☐	☐	☐	☐
People who have long-term health conditions (e.g. HIV, diabetes, heart disease)	☐	☐	☐	☐	☐	☐
People who have weakened immune systems (e.g. tuberculosis, cancer or transplant patients)	☐	☐	☐	☐	☐	☐
People who are in closed settings (e.g. prisons, long-term care facilities)	☐	☐	☐	☐	☐	☐
People who are displaced (e.g. living in refugee camps)	☐	☐	☐	☐	☐	☐
Healthcare/ Social care/ Emergency professionals	☐	☐	☐	☐	☐	☐
People who are homeless	☐	☐	☐	☐	☐	☐

3.c. Which of the following do you think are barriers to implementing a COVID-19 vaccine?

- ☐ Lack of vaccine supply
- ☐ Lack of financial support
- ☐ Lack of policies and guidelines
- ☐ Lack of trained staff
- ☐ Lack of communication between healthcare providers and the public
- ☐ Lack of communication between policy-makers and the public
- ☐ Lack of public support
- ☐ Lack of equipment (e.g. cold chain)
- ☐ Vaccine is manufactured in another country
- ☐ Public fear of vaccine safety
- ☐ Public fear of vaccine effectiveness
- ☐ Socio-cultural beliefs and practices
- ☐ Social stigma
- ☐ Don't know
- ☐ Other

3.c.i. If you selected Other, please specify:

Page 5: Therapeutics

4. Are any medications (drugs) approved for COVID-19 use?

- Yes
- No
- Don't know
- Not applicable (NA) - I don't administer or prescribe medications in my profession

4.a. Which of the following medications/therapies have been approved to prevent/treat COVID-19 in your country?

- Dexamethasone
- Chloroquine
- Hydroxychloroquine
- Remdesivir
- Favipiravir
- Lopinavir-ritonavir (kaletra)
- Interferon beta-1a
- Convalescent plasma
- Natural/traditional treatments
- Other
- Don't know

4.a.i. If you selected Natural/traditional treatments, please specify:

4.a.ii. If you selected Other, please specify:

4.b. How easy do you think it is to provide a COVID-19 oral treatment in terms of practical challenges?

- Very easy
- Easy
- Difficult
- Very difficult
- Don't know

4.b.i. Which of the following do you think are barriers to providing a COVID-19 oral treatment?

- Lack of policies and guidelines
- Lack of communication between policy-makers and healthcare professionals
- Lack of communication between healthcare providers and the public
- Lack of communication between policy-makers and the public
- Lack of public support
- Misinformation (e.g. rumours or myths)
- Socio-cultural beliefs and practices
- Lack of medication supply
- Lack of trained staff
- Social stigma
- Other
- Don't know

4.b.i.a. If you selected Other, please specify:

4.c. How easy do you think it is to provide a COVID-19 treatment via an injection in terms of practical challenges?

- Very easy
- Easy
- Difficult
- Very difficult
- Don't know

4.c.i. Which of the following do you think are barriers to providing a COVID-19 treatment via an injection?

- Lack of policies and guidelines
- Lack of communication between policy-makers and healthcare professionals
- Lack of communication between healthcare providers and the public
- Lack of communication between policy-makers and the public
- Lack of public support
- Misinformation (e.g. rumours or myths)
- Socio-cultural beliefs and practices
- Lack of medication supply
- Lack of equipment to administer treatment (e.g. syringes, needles)
- Lack of trained staff
- Social stigma
- Other
- Don't know

4.c.i.a. If you selected Other, please specify:

4.d. How easy do you think it is to provide a COVID-19 intravenous treatment in terms of practical challenges?

- Very easy
- Easy
- Difficult
- Very difficult
- Don't know

4.d.i. Which of the following do you think are barriers to providing a COVID-19 intravenous treatment?

- Lack of policies and guidelines
- Lack of communication between policy-makers and healthcare professionals
- Lack of communication between healthcare providers and the public
- Lack of communication between policy-makers and the public
- Lack of public support
- Misinformation (e.g. rumours or myths)
- Socio-cultural beliefs and practices
- Lack of medication supply
- Lack of equipment to administer treatment (e.g. infusion pump)
- Lack of trained staff
- Social stigma
- Other
- Don't know

4.d.i.a. If you selected Other, please specify:

4.e. How easy do you think it is to provide a COVID-19 inhaled treatment (e.g. via nebuliser) in terms of practical challenges?

- Very easy
- Easy
- Difficult
- Very difficult
- Don't know

4.e.i. Which of the following do you think are barriers to providing a COVID-19 inhaled treatment?

- ☐ Lack of policies and guidelines
- ☐ Lack of communication between policy-makers and healthcare professionals
- ☐ Lack of communication between healthcare providers and the public
- ☐ Lack of communication between policy-makers and the public
- ☐ Lack of public support
- ☐ Misinformation (e.g. rumours or myths)
- ☐ Socio-cultural beliefs and practices
- ☐ Lack of medication supply
- ☐ Lack of equipment to provide treatment (e.g. nebuliser machine)
- ☐ Lack of trained staff
- ☐ Social stigma
- ☐ Other
- ☐ Don't know

4.e.i.a. If you selected Other, please specify:

4.f. How easy do you think it is to provide blood products (e.g. transfusion of plasma) for COVID-19 treatment in terms of practical challenges?

- ○ Very easy
- ○ Easy
- ○ Difficult
- ○ Very difficult
- ○ Don't know

4.f.i. Which of the following do you think are barriers to providing blood products (e.g. transfusion of plasma) for COVID-19 treatment?

- ☐ Lack of policies and guidelines
- ☐ Lack of communication between policy-makers and healthcare professionals
- ☐ Lack of communication between healthcare providers and the public
- ☐ Lack of communication between policy-makers and the public
- ☐ Lack of public support
- ☐ Misinformation (e.g. rumours or myths)
- ☐ Socio-cultural beliefs and practices
- ☐ Lack of donors
- ☐ Lack of equipment to provide treatment (e.g. intravenous pump)
- ☐ Lack of trained staff
- ☐ Social stigma
- ☐ Lack of blood services
- ☐ Lack of technology infrastructure (e.g. laboratory, cold chain)

- [ ] Other
- [ ] Don't know

4.f.i.a. If you selected Other, please specify:

Page 6: Delivery of Essential Healthcare Services

5. Has the delivery of essential healthcare services been affected?

- Yes
- No
- Don't know

5.a. Which of the following essential healthcare services have been affected?

- Immunisation programmes
- Maternal and Reproductive Health (e.g. facility-based deliveries, antenatal care)
- HIV/AIDs
- Tuberculosis
- Malaria
- Auxiliary services (e.g. laboratory and blood bank services, basic diagnostic imaging)
- Critical inpatient therapies
- Provision of medications and supplies for chronic or communicable diseases
- Care for vulnerable populations
- Emergency care that requires time-sensitive intervention
- Other

5.a.i. If you selected Other, please specify:

5.b. How easy do you think it is for the following essential healthcare services to operate as normal in terms of practical challenges?

	Very easy	Easy	Difficult	Very Difficult	Don't know	Not applicable (NA)
Immunisation programmes						
Maternal and Reproductive Health (e.g. facility-based deliveries, antenatal care)						
HIV/AIDs						
Tuberculosis						
Malaria						
Auxiliary services (e.g. laboratory and blood bank services, basic diagnostic imaging)						
Critical inpatient therapies						
Provision of medications and supplies for chronic or communicable diseases						

Care for vulnerable populations	☐	☐	☐	☐	☐	☐
Emergency care that requires time-sensitive intervention	☐	☐	☐	☐	☐	☐

5.c. Do you think there are barriers to providing essential healthcare services?

- ◯ Yes
- ◯ No
- ◯ Don't know

5.c.i. Which of the following do you think are barriers to providing essential healthcare services?

- ☐ Lack of trained staff
- ☐ Lack of PPE
- ☐ Lack of supply of treatments/vaccines
- ☐ Lack of public support
- ☐ Lack of communication with healthcare providers and the public
- ☐ Lack of communication between policy-makers and healthcare professionals
- ☐ Lack of policies and guidelines
- ☐ Lack of communication with policy-makers and the public
- ☐ Misinformation (e.g. rumours or myths)
- ☐ Other

5.c.i.a. If you selected Other, please specify:

Page 7: Personal Protective Equipment (PPE)

6. Is PPE for COVID-19 being used to perform certain healthcare activities in your place of work?

- ◯ Yes
- ◯ No
- ◯ Don't know

6.a. Which PPE for COVID-19 is being used for the following activities?

	Medical masks	N-95/ FFP2/FFP3 masks	Gowns	Gloves	Aprons	Face shields	Goggles/ glasses	None	Don't know	Not applicable (NA)
Providing direct patient care or assistance to suspected COVID-19 patients	☐	☐	☐	☐	☐	☐	☐	☐	☐	☐
Providing direct patient care or assistance to confirmed COVID-19 patients	☐	☐	☐	☐	☐	☐	☐	☐	☐	☐
Providing direct care to COVID-19 patients in settings where aerosol-generating procedures are frequently performed (e.g. open suctioning, cardiopulmonary resuscitation)	☐	☐	☐	☐	☐	☐	☐	☐	☐	☐
Collecting laboratory samples	☐	☐	☐	☐	☐	☐	☐	☐	☐	☐
Interviewing and assessing persons with or without symptoms (no direct contact/physical distance of at least 1 metre)	☐	☐	☐	☐	☐	☐	☐	☐	☐	☐

6.a.i. If PPE for COVID-19 is being used for Other activities, please specify which activities and what PPE is being used (e.g. medical masks, gowns):

6.b. How easy do you think it is to implement PPE for COVID-19 to perform healthcare activities in terms of practical challenges?

- ○ Very easy
- ○ Easy
- ○ Difficult
- ○ Very difficult
- ○ Don't know

6.b.i. Which of the following do you think are barriers to implementing PPE for COVID-19 to perform healthcare activities?

- ☐ Lack of N-95 / FFP2 / FFP3 masks
- ☐ Lack of medical masks
- ☐ Lack of eye protection equipment
- ☐ Lack of gloves
- ☐ Lack of gowns
- ☐ Lack of policies and guidelines
- ☐ Lack of communication between policy-makers and healthcare professionals
- ☐ Lack of awareness of policies and guidelines among healthcare professionals
- ☐ Insufficient staff training
- ☐ Discomfort of wearing PPE
- ☐ Poor quality of PPE
- ☐ Misinformation (e.g. rumours or myths)
- ☐ Socio-cultural beliefs and practices
- ☐ Social stigma
- ☐ Other
- ☐ Don't know

6.b.i.a. If you selected Other, please specify:

Page 8: About you

7. What is your profession?

- Doctor
- Nurse
- Midwife
- Community health worker
- Other

7.a. If you selected Other, please specify:

8. What type of health facility do you work at/with?

- Community clinic/health post
- Government local/district hospital
- Government referral hospital
- Private community clinic
- Private local/district hospital
- Private referral hospital
- Research institute
- Other

8.a. If you selected Other, please specify:

9. Please tell us which country you have answered the survey questions for:

9.a. If you selected Other, please specify:

10. What is your age?

- 18 - 35
- 35 - 44
- 45 - 54
- 55 - 64
- 65 - 74
- 75 and over
- Prefer not to say

11. What is your gender?

- Male
- Female
- Transgender
- Another gender
- Prefer not to say

Page 9: Additional information

12. Is there anything else you would like us to know?

13. If you are willing to be contacted at a later stage regarding future research work on COVID-19 please provide an email address. In doing so you consent to your details being held anonymously, securely, and separate from other data by the UK-PHRST for 12 months.

Please enter a valid email address.

Page 10: End of survey

We appreciate your time and help completing this survey.

We will share the findings and results to inform outbreak respose to COVID-19 and other diseases in the future through the UK-PHRST Knowledge Hub (https://uk-phrst.tghn.org/) and in publications. You are welcome to join the UKPHRST Knowledge Hub community of practice which brings together public health policymakers and researchers to support translation of data to action (https://uk-phrst.tghn.org/community/).

If you have any questions, please email uk-phrst@lshtm.ac.uk

Thank you.

Key for selection options

9 - Please tell us which country you have answered the survey questions for:

Afghanistan
Albania
Algeria
Andorra
Angola
Antigua and Barbuda
Argentina
Armenia
Australia
Austria
Azerbaijan
Bahamas
Bahrain
Bangladesh
Barbados
Belarus
Belgium
Belize
Benin
Bhutan
Bolivia
Bosnia and Herzegovina
Botswana
Brazil
Brunei Darussalam
Bulgaria
Burkina Faso
Burundi
Cabo Verde
Cambodia
Cameroon
Canada
Central African Republic
Chad
Chile

China
Colombia
Comoros
Congo
Costa Rica
Côte D'Ivoire
Croatia
Cuba
Cyprus
Czech Republic
Democratic People's Republic of Korea
Democratic Republic of the Congo
Denmark
Djibouti
Dominica
Dominican Republic
Ecuador
Egypt
El Salvador
Equatorial Guinea
Eritrea
Estonia
Eswatini
Ethiopia
Fiji
Finland
France
Gabon
Gambia (Republic of The)
Georgia
Germany
Ghana
Greece
Grenada
Guatemala
Guinea
Guinea Bissau
Guyana
Haiti
Honduras
Hungary
Iceland
India
Indonesia
Iran (Islamic Republic of)
Iraq
Ireland
Israel
Italy
Jamaica
Japan
Jordan
Kazakhstan
Kenya
Kiribati
Kuwait

Kyrgyzstan
Lao People’s Democratic Republic
Latvia
Lebanon
Lesotho
Liberia
Libya
Liechtenstein
Lithuania
Luxembourg
Madagascar
Malawi
Malaysia
Maldives
Mali
Malta
Marshall Islands
Mauritania
Mauritius
Mexico
Micronesia (Federated States of)
Monaco
Mongolia
Montenegro
Morocco
Mozambique
Myanmar
Namibia
Nauru
Nepal
Netherlands
New Zealand
Nicaragua
Niger
Nigeria
North Macedonia
Norway
Oman
Palau
Panama
Papua New Guinea
Paraguay
Peru
Philippines
Poland
Portugal
Qatar
Republic of Korea
Republic of Moldova
Romania
Russian Federation
Rwanda
Saint Kitts and Nevis
Saint Lucia
Saint Vincent and the Grenadines
Samoa

San Marino
Sao Tome and Principe
Saudi Arabia
Senegal
Serbia
Seychelles
Sierra Leone
Singapore
Slovakia
Slovenia
Solomon Islands
Somalia
South Africa
South Sudan
Spain
Sri Lanka
Sudan
Suriname
Sweden
Switzerland
Syrian Arab Republic
Tajikistan
Thailand
Timor-Leste
Togo
Tonga
Trinidad and Tobago
Tunisia
Turkey
Turkmenistan
Tuvalu
Uganda
Ukraine
United Arab Emirates
United Kingdom of Great Britain and Northern Ireland
United Republic of Tanzania
United States of America
Uruguay
Uzbekistan
Vanuatu
Venezuela, Bolivarian Republic of
Vietnam
Yemen
Zambia
Zimbabwe
Other

COVID-19 Interventions Survey (Public Health Professionals)

Page 1: Introduction

This survey on public health interventions to respond to COVID-19 is being carried out by researchers from the **UK Public Health Rapid Support Team (UK-PHRST).**

COVID-19 is a challenge worldwide and changing everyone's lives. Public health interventions with restrictions on a scale not seen in modern times are in place; at the same time new methods to support containment are being developed, medications are being tested and in the longer term vaccines may prevent the disease.

This survey aims to gain understanding of public health and healthcare workers' professional assessment of current and future COVID-19 interventions, specifically the practicality of, and barriers to, implementation in their context.

Your participation is important **to share learning to inform the COVID-19 response in your country and in others**. We aim to disseminate the learning from this survey through academic and online platforms to share professional experience worldwide in response to the pandemic.

About This Survey

- This research has received approval from the London School of Hygiene and Tropical Medicine (LSHTM) Ethics Committee.
- The study is for healthcare and public health professionals worldwide.
- This study is anonymous, which means that we will not know who you are, and no information that could indentify you will be used in any report or publication.
- The survey will take around 20 minutes to complete.
- The line at the top of the screen shows your progress through the survey.
- Data will be stored securely at LSHTM's repository.

- This research will comply with the EU General Data Protection Regulation (GDPR) for research data management and storage.
- Summarised results will be shared through the UK-PHRST Knowledge hub and in publications.

We are hoping to get as many healthcare and public health workers worldwide to complete this survey, so please share widely.

For more information please contact uk-phrst@lshtm.ac.uk.

Thank you for your time and help.

---

1. Are you a **public health professional** aged **over 18 years** and **do you agree** to take part in this survey?

- ○ Yes
- ○ No

Page 2: Information about survey questions

Please answer the following questions based on your **professional** opinion with respect to the **country you are most associated with (e.g. where you work or where you live) at the time you complete the survey.**

Page 3: Restrictions on travel: international

2. Are there any restrictions on international travel?

- Yes
- No
- Don't know

2.a. Which restrictions on international travel are in place?

- Entry restrictions: denial of entry from specific countries or any country
- Exit restrictions: denial of exit to specific countries
- Suspended or restricted international flights
- International land borders closure (partial or complete)
- Visa restrictions
- Entry screening and isolation or quarantine
- Exit screening and isolation or quarantine
- Suspended or restricted international ferries or ships
- Other

2.a.i. If you selected Other, please specify:

2.b. How easy do you think it is to implement international entry/exit restrictions in terms of practical challenges?

- Very easy
- Easy
- Difficult
- Very difficult
- Don't know

2.b.i. Which of the following do you think are barriers to implementing international entry/exit restrictions?

- [ ] Lack of enforcement
- [ ] Lack of financial support
- [ ] Lack of trained staff
- [ ] Lack of equipment
- [ ] Lack of policies and guidelines
- [ ] Lack of communication between policy-makers and the public
- [ ] Lack of public support
- [ ] Other
- [ ] Don't know

2.b.i.a. If you selected Other, please specify:

2.c. How easy do you think it is to implement restrictions on international flights, ferries or ships in terms of practical challenges?

- ○ Very easy
- ○ Easy
- ○ Difficult
- ○ Very difficult
- ○ Don't know

2.c.i. Which of the following do you think are barriers to implementing restrictions on international flights, ferries or ships?

- [ ] Lack of enforcement
- [ ] Lack of financial support
- [ ] Lack of trained staff
- [ ] Lack of policies and guidelines
- [ ] Lack of communication between policy-makers and the public
- [ ] Lack of public support
- [ ] Other
- [ ] Don't know

2.c.i.a. If you selected Other, please specify:

2.d. How easy do you think it is to implement entry/exit screening, isolation and quarantine measures in terms of practical challenges?

- ( ) Very easy
- ( ) Easy
- ( ) Difficult
- ( ) Very difficult
- ( ) Don't know

2.d.i. Which of the following do you think are barriers to implementing entry/exit screening, isolation and quarantine measures?

- [ ] Lack of enforcement
- [ ] Lack of financial support
- [ ] Lack of trained staff
- [ ] Lack of equipment
- [ ] Lack of policies and guidelines

- [ ] Lack of communication between policy-makers and the public
- [ ] Lack of public support
- [ ] Other
- [ ] Don't know

2.d.i.a. If you selected Other, please specify:

2.e. How easy do you think it is to implement visa restrictions (e.g. suspension or restrictions on work or travels visas) in terms of practical challenges?

- ○ Very easy
- ○ Easy
- ○ Difficult
- ○ Very difficult
- ○ Don't know

2.e.i. Which of the following do you think are barriers to implementing visa restrictions (e.g. suspension or restrictions on work or travels visas)?

- [ ] Lack of enforcement
- [ ] Lack of financial support
- [ ] Lack of trained staff
- [ ] Lack of policies and guidelines
- [ ] Lack of public support
- [ ] Other
- [ ] Don't know

2.e.i.a. If you selected Other, please specify:

Page 4: Restrictions on travel: in-country (domestic)

3. Are there any restrictions on in-country (domestic) travel?

- ◯ Yes
- ◯ No
- ◯ Don't know

3.a. Which restrictions on in-country (domestic) travel are in place?

- ☐ Denial or restricted entry into other sub-national areas (e.g. states or provinces)
- ☐ Curfew (restricted movement at a particular time)
- ☐ Suspended or restricted movement within the country (e.g. restricted domestic flights, public transport)
- ☐ Non-essential movement restricted (e.g. stay at home order, lockdown)
- ☐ Other

3.a.i. If you selected Other, please specify:

3.b. How easy do you think it is to implement non-essential movement restrictions (e.g. stay at home order, lockdown) in terms of practical challenges?

- ◯ Very easy
- ◯ Easy
- ◯ Difficult
- ◯ Very difficult
- ◯ Don't know

3.b.i. Which of the following do you think are barriers to implementing non-essential movement restrictions (e.g. stay at home order, lockdown)?

- [ ] Lack of enforcement
- [ ] Lack of financial support
- [ ] Lack of trained staff
- [ ] Lack of policies and guidelines
- [ ] Lack of communication between policy-makers and the public
- [ ] Lack of public support
- [ ] Other
- [ ] Don't know

3.b.i.a. If you selected Other, please specify:

3.c. How easy do you think it is to implement denial or restricted entry into other sub-national areas (e.g. states or provinces) / suspended or restricted movement within the country (e.g. restricted domestic flights, public transport) in terms of practical challenges?

- ○ Very easy
- ○ Easy
- ○ Difficult
- ○ Very difficult
- ○ Don't know

3.c.i. Which of the following do you think are barriers to implementing denial or restricted entry into other sub-national areas (e.g. states or provinces) /suspended or restricted movement within the country (e.g. restricted domestic flights, public transport)?

- [ ] Lack of enforcement
- [ ] Lack of trained staff
- [ ] Lack of policies and guidelines

- [ ] Lack of communication between policy-makers and the public
- [ ] Lack of public support
- [ ] Other
- [ ] Don't know

3.c.i.a. If you selected Other, please specify:

Page 5: Restrictions on offices, businesses and institutions

4. Are there any restrictions on offices, institutions or businesses (e.g. partly or fully closed)?

- Yes
- No
- Don't know

4.a. Which restrictions on the following offices, businesses and institutions are in place?

- Offices
- Restaurants
- Bars
- Sports facilities (e.g. clubs, fitness centres, gyms)
- Hotels
- Places of worship
- Cultural centres (e.g. museums, libraries)
- Conference centres
- Food shops/markets
- Entertainment venues (e.g. cinemas)
- Schools (e.g. childcare centres, primary schools, secondary schools)
- Higher Education Institutions (e.g. universities)
- Other

4.a.i. If you selected Other, please specify:

4.b. How easy do you think it is to implement restrictions on offices in terms of practical challenges?

- Very easy
- Easy
- Difficult
- Very difficult
- Don't know

4.b.i. Which of the following do you think are barriers to implementing restrictions on offices?

- Lack of enforcement
- Lack of policies and guidelines
- Lack of communication between policy-makers and the public
- Lack of public support
- Lack of support from employer to work at home
- Lack of financial support for people who have been furloughed (temporary leave)
- Other
- Don't know

4.b.i.a. If you selected Other, please specify:

4.c. How easy do you think it is to implement restrictions on food shops/markets in terms of practical challenges?

- Very easy
- Easy
- Difficult
- Very difficult
- Don't know

4.c.i. Which of the following do you think are barriers to implementing restrictions on food shops/markets?

- [ ] Lack of enforcement
- [ ] Lack of policies and guidelines
- [ ] Lack of communication between policy-makers and the public
- [ ] Lack of public support
- [ ] Lack of financial support for food shop/market owners
- [ ] Lack of financial support for people who have been furloughed (put on temporary leave)
- [ ] Other
- [ ] Don't know

4.c.i.a. If you selected Other, please specify:

4.d. How easy do you think it is to implement restrictions on places of worship in terms of practical challenges?

- ( ) Very easy
- ( ) Easy
- ( ) Difficult
- ( ) Very difficult
- ( ) Don't know

4.d.i. Which of the following do you think are barriers to implementing restrictions on places of worship?

- [ ] Lack of enforcement
- [ ] Lack of policies and guidelines
- [ ] Lack of communication between policy-makers and the public
- [ ] Lack of public support
- [ ] Other
- [ ] Don't know

4.d.i.a. If you selected Other, please specify:

4.e. How easy do you think it is to implement restrictions on schools or higher education instituitions in terms of practical challenges?

- Very easy
- Easy
- Difficult
- Very difficult
- Don't know

4.e.i. Which of the following do you think are barriers to implementing restrictions on schools or higher eductation institutes?

- [ ] Lack of enforcement
- [ ] Lack of policies and guidelines
- [ ] Lack of communication between policy-makers and the public
- [ ] Lack of public support
- [ ] Lack of remote/distance learning support (e.g. limited or no access to remote education resources, laptops, internet)
- [ ] Lack of childcare support

- [ ] Other
- [ ] Don't know

4.e.i.a. If you selected Other, please specify:

4.f. How easy do you think it is to implement restrictions on bars, hotels and restaurants in terms of practical challenges?

- Very easy
- Easy
- Difficult
- Very difficult
- Don't know

4.f.i. Which of the following do you think are barriers to implementing restrictions on bars, restaurants and hotels?

- [ ] Lack of enforcement
- [ ] Lack of policies and guidelines
- [ ] Lack of communication between policy-makers and the public
- [ ] Lack of public support
- [ ] Lack of financial support for owners of bars, restaurants and hotels
- [ ] Other
- [ ] Don't know

4.f.i.a. If you selected Other, please specify:

4.g. How easy do you think it is to implement restrictions on entertainment, conference, sport and cultural centres in terms of practical challenges?

- Very easy
- Easy
- Difficult
- Very difficult
- Don't know

4.g.i. Which of the following do you think are barriers to implementing restrictions on entertainment, conference, sport and cultural centres ?

- Lack of enforcement
- Lack of policies and guidelines
- Lack of communication between policy-makers and the public
- Lack of public support
- Lack of financial support for owners of restrictions on entertainment, conference, sport and cultural centres
- Other
- Don't know

4.g.i.a. If you selected Other, please specify:

Page 6: Restrictions on gatherings of people

5. Are there any restrictions on gatherings of people?

- Yes
- No
- Don't know

5.a. Which restrictions on gatherings of people are in place?

- Private gatherings at home
- Private gatherings outside the home (e.g. funerals)
- Public gatherings outside the home
- Mass gatherings (e.g. conferences)
- Other

5.a.i. If you selected Other, please specify:

5.b. How easy do you think it is to implement restrictions on private gatherings at home in terms of practical challenges?

- Very easy
- Easy
- Difficult
- Very difficult
- Don't know

5.b.i. Which of the following do you think are barriers to implementing restrictions on private gatherings at home?

- [ ] Lack of enforcement
- [ ] Lack of policies and guidelines
- [ ] Lack of communication between policy-makers and the public
- [ ] Lack of public support
- [ ] Other
- [ ] Don't know

5.b.i.a. If you selected Other, please specify:

5.c. How easy do you think it is to implement restrictions on private gatherings outside the home (e.g. funerals) in terms of practical challenges?

- ◯ Very easy
- ◯ Easy
- ◯ Difficult
- ◯ Very difficult
- ◯ Don't know

5.c.i. Which of the following do you think are barriers to implementing restrictions on private gatherings outisde the home (e.g. funerals)?

- [ ] Lack of enforcement
- [ ] Lack of policies and guidelines
- [ ] Lack of communication between policy-makers and the public
- [ ] Lack of public support
- [ ] Other
- [ ] Don't know

5.c.i.a. If you selected Other, please specify:

5.d. How easy do you think it is to implement restrictions on public gatherings outside the home in terms of practical challenges?

- Very easy
- Easy
- Difficult
- Very difficult
- Don't know

5.d.i. Which of the following do you think are barriers to implementing restrictions on public gatherings outisde the home?

- Lack of enforcement
- Lack of policies and guidelines
- Lack of communication between policy-makers and the public
- Lack of public support
- Socio-cultural practices and beliefs
- Other
- Don't know

5.d.i.a. If you selected Other, please specify:

5.e. How easy do you think it is to implement restrictions on mass gatherings (e.g. conferences) in terms of practical challenges?

- Very easy
- Easy
- Difficult
- Very difficult
- Don't know

5.e.i. Which of the following do you think are barriers to implementing restrictions on mass gatherings (e.g. conferences)?

- Lack of enforcement
- Lack of policies and guidelines
- Lack of communication between policy-makers and the public
- Lack of public support
- Socio-cultural practices and beliefs
- Lack of resources to support mass gathering remotely (e.g. technology infrastructure)
- Other
- Don't know

5.e.i.a. If you selected Other, please specify:

Page 7: Individual measures

6. Are there any individual measures in place (e.g. handwashing, facemasks)?

- ○ Yes
- ○ No
- ○ Don't know

6.a. Which individual measures are in place?

- ☐ Handwashing
- ☐ Respiratory etiquette (e.g. covering mouth and nose with a tissue when coughing or sneezing)
- ☐ Face masks
- ☐ Face coverings
- ☐ Physical distancing
- ☐ Temperature monitoring
- ☐ Other

6.a.i. If you selected Other, please specify:

6.b. How easy do you think it is to implement handwashing in terms of practical challenges?

- ○ Very easy
- ○ Easy
- ○ Difficult
- ○ Very difficult
- ○ Don't know

6.b.i. Which of the following do you think are barriers to implementing handwashing?

- ☐ Lack of enforcement
- ☐ Lack of policies and guidelines
- ☐ Lack of communication between policy-makers and the public
- ☐ Lack of communication between healthcare providers and the public
- ☐ Lack of public support
- ☐ Lack of resources (e.g. handwashing areas, water, soap, alcohol rub)
- ☐ Social stigma
- ☐ Misinformation (e.g. rumours or myths)
- ☐ Socio-cultural beliefs and practices
- ☐ Other
- ☐ Don't know

6.b.i.a. If you selected Other, please specify:

6.c. How easy do you think it is to implement respiratory etiquette (e.g. covering mouth and nose with a bent elbow or tissue when coughing or sneezing) in terms of practical challenges?

- ○ Very easy
- ○ Easy
- ○ Difficult
- ○ Very difficult
- ○ Don't know

6.c.i. Which of the following do you think are barriers to implementing respiratory etiquette (e.g. covering mouth and nose with a bent elbow or tissue when coughing or

sneezing)?

- Lack of enforcement
- Lack of policies and guidelines
- Lack of communication between policy-makers and the public
- Lack of communication between healthcare providers and the public
- Lack of public support
- Social stigma
- Misinformation (e.g. rumours or myths)
- Socio-cultural beliefs and practices
- Other
- Don't know

6.c.i.a. If you selected Other, please specify:

6.d. How easy do you think it is to implement face masks/ face coverings in terms of practical challenges?

- Very easy
- Easy
- Difficult
- Very difficult
- Don't know

6.d.i. Which of the following do you think are barriers to implementing face masks/ face coverings?

- Lack of enforcement

- ☐ Lack of policies and guidelines
- ☐ Lack of communication between policy-makers and the public
- ☐ Lack of communication between healthcare providers and the public
- ☐ Lack of public support
- ☐ Lack of resources (e.g. face masks/ face coverings)
- ☐ Social stigma
- ☐ Misinformation (e.g. rumours or myths)
- ☐ Socio-cultural beliefs and practices
- ☐ Other
- ☐ Don't know

6.d.i.a. If you selected Other, please specify:

6.e. How easy do you think it is to implement physical distancing in terms of practical challenges?

- ○ Very easy
- ○ Easy
- ○ Difficult
- ○ Very difficult
- ○ Don't know

6.e.i. Which of the following do you think are barriers to implementing physical distancing?

- ☐ Lack of enforcement
- ☐ Lack of policies and guidelines
- ☐ Lack of communication between policy-makers and the public

- [ ] Lack of communication between healthcare providers and the public
- [ ] Lack of public support
- [ ] Social stigma
- [ ] Misinformation (e.g. rumours or myths)
- [ ] Socio-cultural beliefs and practices
- [ ] Not possible to maintain physical distance at workplace due to the nature of the work
- [ ] Other
- [ ] Don't know

6.e.i.a. If you selected Other, please specify:

6.f. How easy do you think it is to implement temperature monitoring in terms of practical challenges?

- ( ) Very easy
- ( ) Easy
- ( ) Difficult
- ( ) Very difficult
- ( ) Don't know

6.f.i. Which of the following do you think are barriers to implementing temperature monitoring?

- [ ] Lack of enforcement
- [ ] Lack of policies and guidelines
- [ ] Lack of communication between policy-makers and the public
- [ ] Lack of communication between healthcare providers and the public

- [ ] Lack of public support
- [ ] Lack of equipment
- [ ] Social stigma
- [ ] Misinformation (e.g. rumours or myths)
- [ ] Socio-cultural beliefs and practices
- [ ] Other
- [ ] Don't know

6.f.i.a. If you selected Other, please specify:

Page 8: Contact tracing

7. Is contact tracing being conducted?

- Yes
- No
- Don't know

7.a. Which contact tracing methods are being used?

- Physical contact tracing (e.g. conducted by people)
- Mobile phone applications (apps) (e.g. Mobile phone global positioning system (GPS))
- Closed-circuit television (CCTV)
- Electronic tags
- Bank card transactions
- Other
- Don't know

7.a.i. If you selected Other, please specify:

7.b. How easy do you think it is to implement physical contact tracing methods (e.g. in-person visits, by phone) in terms of practical challenges?

- Very easy
- Easy
- Difficult
- Very difficult
- Don't know

7.b.i. Which of the following do you think are barriers to implementing physical contact tracing methods (e.g. in-person visits, by phone)?

- [ ] Lack of enforcement
- [ ] Lack of policies and guidelines
- [ ] Lack of communication between policy-makers and the public
- [ ] Lack of communication between healthcare providers and the public
- [ ] Lack of public support
- [ ] Lack of trained staff
- [ ] Lack of technology infrastructure
- [ ] Privacy laws
- [ ] Social stigma
- [ ] Misinformation (e.g. rumours or myths)
- [ ] Socio-cultural beliefs and practices
- [ ] Other
- [ ] Don't know

7.b.i.a. If you selected Other, please specify:

7.c. How easy do you think it is to implement mobile phone contact tracing apps / GPS-based tracing in terms of practical challenges?

- ○ Very easy
- ○ Easy
- ○ Difficult
- ○ Very difficult
- ○ Don't know

7.c.i. Which of the following do you think are barriers to implementing mobile phone contact tracing apps / GPS-based tracing?

- ☐ Lack of enforcement
- ☐ Lack of policies and guidelines
- ☐ Lack of communication between policy-makers and the public
- ☐ Lack of communication between healthcare providers and the public
- ☐ Lack of public support
- ☐ Lack of trained staff
- ☐ Lack of technology infrastructure
- ☐ Privacy laws
- ☐ Social stigma
- ☐ Misinformation (e.g. rumours or myths)
- ☐ Socio-cultural beliefs and practices
- ☐ Other
- ☐ Don't know

7.c.i.a. If you selected Other, please specify:

7.d. How easy do you think it is to implement closed-circuit television (CCTV)/ electronic tags as contact tracing methods in terms of practical challenges?

- ○ Very easy
- ○ Easy
- ○ Difficult
- ○ Very difficult
- ○ Don't know

7.d.i. Which of the following do you think are barriers to implemening closed-circuit television (CCTV)/ electronic tags as contact tracing methods?

- [ ] Lack of enforcement
- [ ] Lack of policies and guidelines
- [ ] Lack of communication between policy-makers and the public
- [ ] Lack of communication between healthcare providers and the public
- [ ] Lack of public support
- [ ] Lack of trained staff
- [ ] Lack of technology infrastructure
- [ ] Privacy laws
- [ ] Social stigma
- [ ] Misinformation (e.g. rumours or myths)
- [ ] Socio-cultural beliefs and practices
- [ ] Other
- [ ] Don't know

7.d.i.a. If you selected Other, please specify:

7.e. How easy do you think it is to implement contact tracing methods using peoples' bank card transactions in terms of practical challenges?

- ( ) Very easy
- ( ) Easy
- ( ) Difficult
- ( ) Very difficult
- ( ) Don't know

7.e.i. Which of the following do you think are barriers to implemening contact tracing methods using peoples' bank card transactions?

- [ ] Lack of enforcement
- [ ] Lack of policies and guidelines
- [ ] Lack of communication between policy-makers and the public
- [ ] Lack of communication between healthcare providers and the public
- [ ] Lack of public support
- [ ] Lack of trained staff
- [ ] Lack of technology infrastructure
- [ ] Privacy laws
- [ ] Social stigma
- [ ] Misinformation (e.g. rumours or myths)
- [ ] Socio-cultural beliefs and practices
- [ ] Other
- [ ] Don't know

7.e.i.a. If you selected Other, please specify:

Page 9: Shielding

Shielding is a measure to protect vulnerable people from coming into contact with COVID-19, by restricting interactions between them and others.

8. Are any population groups being shielded?

- ○ Yes
- ○ No
- ○ Don't know

8.a. Which population groups are being shielded?

- ☐ Pregnant women
- ☐ Older people
- ☐ People who have long-term health conditions (e.g. HIV, diabetes, heart disease)
- ☐ People who have weakened immune systems (e.g. tuberculosis (TB), cancer or transplant patients)
- ☐ Other
- ☐ Don't know

8.a.i. If you selected Other, please specify:

8.b. How easy do you think it is to implement shielding measures for pregnant women in terms of practical challenges?

- ○ Very easy
- ○ Easy

- Difficult
- Very difficult
- Don't know

8.b.i. Which of the following do you think are barriers to implementing shielding measures for pregnant women?

- Lack of enforcement
- Lack of policies and guidelines
- Lack of communication between policy-makers and the public
- Lack of communication between healthcare providers and the public
- Lack of public support
- Lack of home support for pregnant women (e.g. medical care, supply of food/medications)
- Lack of financial support for pregnant women
- Social stigma
- Misinformation (e.g. rumours or myths)
- Socio-cultural beliefs and practices
- Other
- Don't know

8.b.i.a. If you selected Other, please specify:

8.c. How easy do you think it is to implement shielding measures for older people in terms of practical challenges?

- Very easy
- Easy

- Difficult
- Very difficult
- Don't know

8.c.i. Which of the following do you think are barriers to implementing shielding measures for older people?

- Lack of enforcement
- Lack of policies and guidelines
- Lack of communication between policy-makers and the public
- Lack of communication between healthcare providers and the public
- Lack of public support
- Lack of home support for older people (e.g. medical care, supply of food/medications)
- Social stigma
- Misinformation (e.g. rumours or myths)
- Socio-cultural beliefs and practices
- Other
- Don't know

8.c.i.a. If you selected Other, please specify:

8.d. How easy do you think it is to implement shielding measures for people with long-term conditions or those with weakened immune systems in terms of practical challenges?

- Very easy
- Easy

- Difficult
- Very difficult
- Don't know

8.d.i. Which of the following do you think are barriers to implementing shielding measures for people with long-term conditions or those with weakened immune systems?

- [ ] Lack of enforcement
- [ ] Lack of policies and guidelines
- [ ] Lack of communication between policy-makers and the public
- [ ] Lack of communication between policy-makers and healthcare providers
- [ ] Lack of communication between healthcare providers and the public
- [ ] Lack of public support
- [ ] Lack of home support for people with long-term conditions or those with weakened immune systems (e.g. medical care, supply of food/medications)
- [ ] Social stigma
- [ ] Misinformation (e.g. rumours or myths)
- [ ] Socio-cultural beliefs and practices
- [ ] Other
- [ ] Don't know

8.d.i.a. If you selected Other, please specify:

Page 10: Vaccines

The following questions refer to **vaccine use**, assuming a safe and effective vaccine for COVID-19 can be developed.

9. Do you think a vaccine, if developed, should be introduced to prevent COVID-19?

- Yes
- No
- Don't know

9.a. Do you think a vaccine should be prioritised for certain popualtion groups (e.g. pregnant women, healthcare workers, older people)?

- Yes
- No – everyone should be vaccinated
- Don't know

9.a.i. Which of the following population groups do you think a vaccine should be prioritised for? (please select up to 3 groups)

- Infants
- School age children
- Pregnant women
- People who are working
- People who are carers for dependents
- Older people
- People who have long-term health conditions (e.g. diabetes, heart disease)
- People who have weakened immune systems (e.g. cancer or transplant patients)
- People who are in closed settings (e.g. prisons, long-term care facilities)
- People who are displaced (e.g. living in refugee camps)
- Healthcare/ Social care/ Emergency professionals
- Homeless people

- [ ] Don't know
- [ ] Other

9.a.i.a. If you selected Other, please specify:

9.b. How easy do you think it is to provide a COVID-19 vaccine to these population groups in terms of practical challenges?

	Very easy	Easy	Difficult	Very difficult	Don't know	Not applicable (NA)
Infants	☐	☐	☐	☐	☐	☐
School age children	☐	☐	☐	☐	☐	☐
Pregnant women	☐	☐	☐	☐	☐	☐
People who are working	☐	☐	☐	☐	☐	☐
People who are carers for dependents	☐	☐	☐	☐	☐	☐
Older people	☐	☐	☐	☐	☐	☐
People who have long-term health conditions (e.g. HIV, diabetes, heart disease)	☐	☐	☐	☐	☐	☐

People who have weakened immune systems (e.g. tuberculosis, cancer or transplant patients)	☐	☐	☐	☐	☐	☐
People who are in closed settings (e.g. prisons, long-term care facilities)	☐	☐	☐	☐	☐	☐
People who are displaced (e.g. living in refugee camps)	☐	☐	☐	☐	☐	☐
Healthcare/ Social care/ Emergency professionals	☐	☐	☐	☐	☐	☐
People who are homeless	☐	☐	☐	☐	☐	☐

9.c. Which of the following do you think are barriers to implementing a COVID-19 vaccine?

- ☐ Lack of vaccine supply
- ☐ Lack of financial support
- ☐ Lack of policies and guidelines
- ☐ Lack of trained staff
- ☐ Lack of communication between healthcare providers and the public
- ☐ Lack of communication between policy-makers and the public
- ☐ Lack of public support

- ☐ Lack of equipment (e.g. cold chain)
- ☐ Vaccine is manufactured in another country
- ☐ Public fear of vaccine safety
- ☐ Public fear of vaccine effectiveness
- ☐ Socio-cultural beliefs and practices
- ☐ Social stigma
- ☐ Other
- ☐ Don't know

9.c.i. If you selected Other, please specify:

Page 11: About you

10. What type of institution do you work for?

- ☐ Government
- ☐ Non-governmental or not-for-profit agency
- ☐ Research
- ☐ Higher Education
- ☐ Multilateral organisation
- ☐ Other

10.a. If you selected Other, please specify:

11. What level do you work at?

- ☐ Community/District
- ☐ County/Regional
- ☐ National
- ☐ International
- ☐ Other

11.a. If you selected Other, please specify:

12. Please tell us which country you have answered the survey questions for:

12.a. If you selected Other, please specify:

13. What is your age?

- 18 - 35
- 35 - 44
- 45 - 54
- 55 - 64
- 65 - 74
- 75 and over
- Prefer not to say

14. What is your gender?

- Male
- Female
- Transgender
- Another gender
- Prefer not to say

Page 12: Additional information

15. Is there anything else you would like us to know?

16. If you are willing to be contacted at a later stage regarding future research work on COVID-19 please provide an email address. In doing so you consent to your details being held anonymously, securely and separate from other data by the UK-PHRST for 12 months.

Page 13: End of survey

We appreciate your time and help completing this survey.

We will share the findings and results to inform outbreak respose to COVID-19 and other diseases in the future through the UK-PHRST Knowledge Hub (https://uk-phrst.tghn.org/) and in publications. You are welcome to join the UKPHRST Knowledge Hub community of practice which brings together public health policymakers and researchers to support translation of data to action (https://uk-phrst.tghn.org/community/).

If you have any questions, please email uk-phrst@lshtm.ac.uk

Thank you.

Key for selection options

12 - Please tell us which country you have answered the survey questions for:

Afghanistan
Albania
Algeria
Andorra
Angola
Antigua and Barbuda
Argentina
Armenia
Australia
Austria
Azerbaijan
Bahamas
Bahrain
Bangladesh
Barbados
Belarus
Belgium
Belize

Benin
Bhutan
Bolivia
Bosnia and Herzegovina
Botswana
Brazil
Brunei Darussalam
Bulgaria
Burkina Faso
Burundi
Cabo Verde
Cambodia
Cameroon
Canada
Central African Republic
Chad
Chile
China
Colombia
Comoros
Congo
Costa Rica
Côte D'Ivoire
Croatia
Cuba
Cyprus
Czech Republic
Democratic People's Republic of Korea
Democratic Republic of the Congo
Denmark
Djibouti
Dominica
Dominican Republic
Ecuador
Egypt
El Salvador
Equatorial Guinea
Eritrea
Estonia
Eswatini

Ethiopia
Fiji
Finland
France
Gabon
Gambia (Republic of The)
Georgia
Germany
Ghana
Greece
Grenada
Guatemala
Guinea
Guinea Bissau
Guyana
Haiti
Honduras
Hungary
Iceland
India
Indonesia
Iran (Islamic Republic of)
Iraq
Ireland
Israel
Italy
Jamaica
Japan
Jordan
Kazakhstan
Kenya
Kiribati
Kuwait
Kyrgyzstan
Lao People’s Democratic Republic
Latvia
Lebanon
Lesotho
Liberia
Libya

Liechtenstein
Lithuania
Luxembourg
Madagascar
Malawi
Malaysia
Maldives
Mali
Malta
Marshall Islands
Mauritania
Mauritius
Mexico
Micronesia (Federated States of)
Monaco
Mongolia
Montenegro
Morocco
Mozambique
Myanmar
Namibia
Nauru
Nepal
Netherlands
New Zealand
Nicaragua
Niger
Nigeria
North Macedonia
Norway
Oman
Palau
Panama
Papua New Guinea
Paraguay
Peru
Philippines
Poland
Portugal
Qatar

Republic of Korea
Republic of Moldova
Romania
Russian Federation
Rwanda
Saint Kitts and Nevis
Saint Lucia
Saint Vincent and the Grenadines
Samoa
San Marino
Sao Tome and Principe
Saudi Arabia
Senegal
Serbia
Seychelles
Sierra Leone
Singapore
Slovakia
Slovenia
Solomon Islands
Somalia
South Africa
South Sudan
Spain
Sri Lanka
Sudan
Suriname
Sweden
Switzerland
Syrian Arab Republic
Tajikistan
Thailand
Timor-Leste
Togo
Tonga
Trinidad and Tobago
Tunisia
Turkey
Turkmenistan
Tuvalu

Uganda
Ukraine
United Arab Emirates
United Kingdom of Great Britain and Northern Ireland
United Republic of Tanzania
United States of America
Uruguay
Uzbekistan
Vanuatu
Venezuela, Bolivarian Republic of
Vietnam
Yemen
Zambia
Zimbabwe
Other
